# A Coordinated Interdependent Protein Circuitry Stabilizes the Kinetochore Ensemble to Protect CENP-A in the Human Pathogenic Yeast *Candida albicans*

**DOI:** 10.1371/journal.pgen.1002661

**Published:** 2012-04-19

**Authors:** Jitendra Thakur, Kaustuv Sanyal

**Affiliations:** Molecular Mycology Laboratory, Molecular Biology and Genetics Unit, Jawaharlal Nehru Centre for Advanced Scientific Research, Bangalore, India; Duke University, United States of America

## Abstract

Unlike most eukaryotes, a kinetochore is fully assembled early in the cell cycle in budding yeasts *Saccharomyces cerevisiae* and *Candida albicans*. These kinetochores are clustered together throughout the cell cycle. Kinetochore assembly on point centromeres of *S. cerevisiae* is considered to be a step-wise process that initiates with binding of inner kinetochore proteins on specific centromere DNA sequence motifs. In contrast, kinetochore formation in *C. albicans*, that carries regional centromeres of 3–5 kb long, has been shown to be a sequence independent but an epigenetically regulated event. In this study, we investigated the process of kinetochore assembly/disassembly in *C. albicans*. Localization dependence of various kinetochore proteins studied by confocal microscopy and chromatin immunoprecipitation (ChIP) assays revealed that assembly of a kinetochore is a highly coordinated and interdependent event. Partial depletion of an essential kinetochore protein affects integrity of the kinetochore cluster. Further protein depletion results in complete collapse of the kinetochore architecture. In addition, GFP-tagged kinetochore proteins confirmed similar time-dependent disintegration upon gradual depletion of an outer kinetochore protein (Dam1). The loss of integrity of a kinetochore formed on centromeric chromatin was demonstrated by reduced binding of CENP-A and CENP-C at the centromeres. Most strikingly, Western blot analysis revealed that gradual depletion of any of these essential kinetochore proteins results in concomitant reduction in cellular protein levels of CENP-A. We further demonstrated that centromere bound CENP-A is protected from the proteosomal mediated degradation. Based on these results, we propose that a coordinated interdependent circuitry of several evolutionarily conserved essential kinetochore proteins ensures integrity of a kinetochore formed on the foundation of CENP-A containing centromeric chromatin.

## Author Summary

The kinetochore, a macromolecular protein complex that assembles on centromere DNA, interacts with spindle microtubules to mediate faithful chromosome segregation. The sequence of centromere DNA, which varies from 125 bp long point centromere in *Saccharomyces cerevisiae* to a few Mb long regional centromeres in humans, does not show any conservation across species. Kinetochore proteins, however, share a higher degree of conservation in amino acid sequence. Intriguingly, kinetochore assembly has been shown to be species-specific, although specialized centromeric chromatin is always formed by the centromeric histone CENP-A. We investigated this process of kinetochore assembly on epigenetically determined regional centromeres in the pathogenic budding yeast *Candida albicans*. Here we established that a coordinated interdependent assembly of several essential evolutionarily conserved kinetochore proteins ensures integrity of a functional kinetochore. Depletion of an essential kinetochore protein leads to total collapse of the kinetochore architecture. We observe that kinetochore disintegration precedes kinetochore collapse. Finally, we prove that kinetochore integrity keeps centromeric chromatin intact and protects CENP-A molecules from proteasomal mediated degradation.

## Introduction

The centromeric histone CENP-A acts as the epigenetic mark of a functional centromere from yeast to humans [1]. As a histone H3 variant, CENP-A replaces canonical histone H3 to mark specialized centromeric chromatin by a mechanism that remains largely unknown. While CENP-A deposition occurs in a sequence-dependent manner in point centromeres of certain budding yeasts including *S. cerevisiae* [2], [3], its recruitment to regional centromeres of most other eukaryotes is not strictly sequence dependent [1], [3]–[5]. Several lines of evidence suggest that the composition of nucleosomes that form centromeric chromatin may vary from species to species [6]–[10]. Even the hierarchy of events that assembles and stabilizes a complex macromolecular kinetochore (KT) structure on unusual centromeric chromatin, whether universal or species-specific, remains unclear. CENP-A is believed to be the initiator of the process of KT formation [1], [11]. Localization of most KT proteins is regulated by CENP-A [12]–[15]. However, a few proteins such as Ndc10 and Scm3 in *S. cerevisiae* [6], [16], Mis12/Mtw1 in *C. albicans* [17], Mis6, Mis16-Mis18 complex and Ams2 in *Schizosaccharomyces pombe* [18], [19], and CENP-H-I complex in humans [20] have been shown to regulate CENP-A localization.

Although the structure of metazoan KTs can be visualized under microscope, the exact nature of the KT architecture is difficult to ascertain in yeasts due to small size of these cells [21]. However, based on presence of functional homologs of several KT proteins, and their genetic as well as biochemical interaction in various eukaryotes, it is presumed that the three-layered KT structure is evolutionarily conserved from yeasts to humans. A KT helps in bridging the mitotic spindle to centromere (*CEN*) DNA to ensure faithful chromosome segregation during mitosis and meiosis. KT proteins exist as sub-complexes that assemble on *CEN* DNA [22]–[24]. In humans, inner (CENP-A, -B, -C, -H and –I) and middle (the Spc105 complex and the Mis12 complex) KT proteins are associated constitutively with *CEN* DNA [25] but outer KT proteins (such as the Ndc80 complex and the Ska1 complex) which help in KT-microtubule (MT) interaction are generally localized to KTs only during mitosis [26], [27]. In contrast, KTs are fully assembled in *S. cerevisiae* early in the cell cycle. The fungal specific Dam1 complex, an outer KT protein complex in budding yeast *S. cerevisiae*, remains associated with KTs throughout the cell cycle [28]–[30].

One of the less understood features of budding yeast KTs is that they are clustered together throughout the cell cycle [31]. A recent study, using chromosome conformation capture-on-chip (4C), clearly demonstrates that all chromosomes cluster via centromeres at one pole of the nucleus in *S. cerevisiae* suggesting interchromosomal cross-talks through inter-KT interaction [32]. KT clustering has been shown to be important for centromere function in *S. cerevisiae* as KTs are found to be declustered in *ndc10*, *ame2* and *nuf2* KT mutants [31], [33]. Interestingly, KTs are clustered only during interphase but not in mitosis in fission yeast *S. pombe* [34]. In metazoans KTs are never clustered [35].

With the notable exception of holocentric chromosomes of nematodes and aphids where KTs are formed across the entire length of a chromosome [36], only one KT is formed on each chromosome in all other organisms studied till date. A functional KT can even assemble on non-centromeric DNA to form a neocentromere in certain organisms when a native centromere is deleted or inactivated [37]–[40]. Thus there must be an underlying active mechanism to prevent formation of centromeric chromatin on neocentromeric loci when the native centromere is functional. CENP-A at non-centromeric locations is targeted for proteasomal degradation in *Drosophila melanogaster* and *S. cerevisiae* [41]–[43]. Thus ectopic CENP-A is destabilized to prevent multiple kinetochore formation although the exact cellular signal that distinguishes CENP-A molecules present at the native centromere from those ectopically localized could not be determined.

*Candida albicans*, a pathogenic budding yeast, that causes candidiasis in humans, carries 3–5 kb long unique centromeric chromatin on each of its eight chromosomes. There are 4 CENP-A molecules but only one MT binds to a KT in *C. albicans* [44]. Centromeric regions have been shown to have unusual chromatin structure [45] and histone H3 molecules are replaced by CENP-A at *CEN*s (K. Sanyal, unpublished) in this organism. Moreover, CENP-A deposition on *CEN*s has been shown to be epigenetically regulated [45]–[47]. We have previously cloned and characterized several evolutionarily conserved KT proteins including CENP-A/Cse4 [48], CENP-C/Mif2 [46], Mis12/Mtw1 [17] and the Dam1 complex subunits [49] in *C. albicans* and shown that each of these proteins is essential in a KT-MT-mediated process of chromosome segregation.

In the present work, we studied localization interdependence of KT proteins that govern KT integrity including stability of CENP-A. Our results reveal that the KT architecture is stabilized in a coordinated interdependent manner by individual components of the KT in *C. albicans*. Most strikingly, we provide evidence that stability of CENP-A molecules is determined by integrity of the KTs. This is the first demonstration, to our knowledge, of how an interdependent circuitry of several KT proteins helps stabilizing CENP-A at a functional KT.

## Results

### Centromeric localization of CENP-A is dependent upon various kinetochore proteins in *C. albicans*

**Figure 1:**
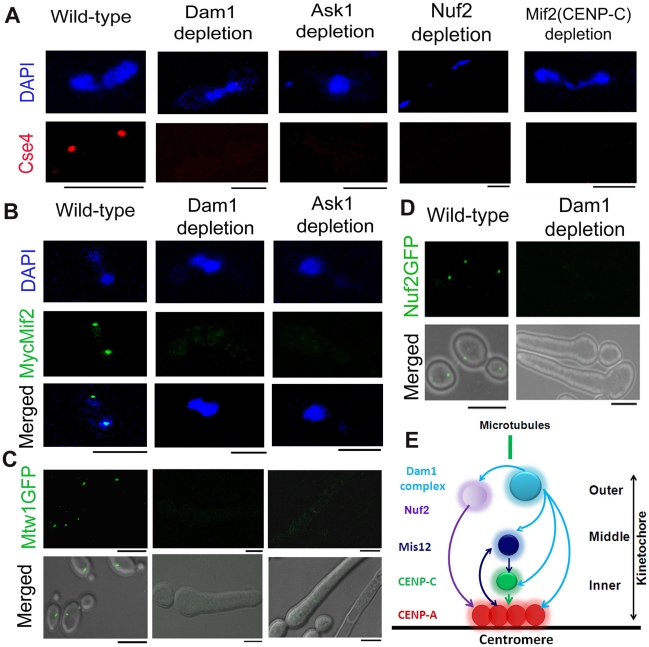
The process of kinetochore assembly is interdependent and coordinated in *C. albicans*. (A) Mutant strain J102 (*MET3*pr*DAM1/dam1*), J104 (*MET3*pr*ASK1/ask1*), YJB12326 (*MET3*pr*NUF2/nuf2*) or CAMB2 (*PCK1*pr*MycMIF2/mif2*) was grown for 8 h under non-permissive conditions to deplete Dam1, Ask1, Nuf2 or Mif2 respectively along with wild-type BWP17. Cells from each culture were fixed and stained with DAPI and anti-Cse4 antibodies. CENP-A/Cse4 signals, visible in wild-type cells, were undetected in Dam1, Ask1, Nuf2 or Mif2 depleted cells. (B) Wild-type J125 (*PCK1*pr*MycMIF2/MIF2*), *dam1* mutant J123 (*MET3*pr*DAM1/dam1 PCK1*pr*MycMIF2/MIF2*) or *ask1* mutant J124 (*MET3*pr*ASK1/ask1 MIF2/PCK1*pr*MycMIF2*) was grown for 8 h under non-permissive conditions (+Cys +Met +Suc) of the *MET3* promoter, fixed and stained with DAPI, and anti-Myc antibodies. MycMif2 signals, clearly visible in wild-type, were undetected in Dam1- or Ask1-depleted cells. (C) Wild-type YJB10695 (*MTW1GFP/MTW1*), *dam1* mutant J122 (*MET3*pr*DAM1/dam1 MTW1GFP/MTW1*), or *ask1* mutant J120 (*MET3*pr*ASK1/ask1 MTW1GFP/MTW1*) cells were grown under non-permissive conditions of the *MET3* promoter (+Cys +Met) for 8 h and examined for GFP signals by confocal microscopy. Bright Mtw1-GFP dot-like signals were observed in wild-type cells but no Mtw1-GFP signals were detected in Dam1- or Ask-depleted cells. (D) YJB12289 (*MET3*pr*DAM1/dam1 NUF2-GFP/NUF2*) cells grown in conditions where Dam1 is either expressed (−Cys −Met; wild-type) or repressed (+Cys +Met; Dam1 depleted) for 8 h were examined for GFP signals by confocal microscopy. Bright Nuf2-GFP dot-like signals were observed in wild-type cells but not in Dam1-depleted cells. (E) Schematic showing localization dependence of evolutionarily conserved KT proteins in *C. albicans*. Arrows show localization dependence observed in this study or reported previously [17]. Bars, 5 µm.

In order to understand the process of KT assembly in *C. albicans*, we studied localization dependence of various essential KT proteins that are evolutionarily conserved. To achieve this, we utilised conditional mutant strains carrying KT proteins under control of the *MET3* or *PCK1* promoter. The *MET3* promoter is repressed in presence of cysteine (Cys) and methionine (Met) [50] while the *PCK1* promoter is repressed when glucose (Glu) is used as the carbon source [51]. We depleted each of Dam1, Ask1, Spc19 or Dad2 - subunits of an essential outer KT protein complex, the Dam1 complex, in J102 (*MET3*pr*DAM1/dam1*), J104 (*MET3*pr*ASK/ask1*), J106 (*MET3*pr*SPC19/spc19*) or J108 (*PCK1*pr*DAD2/dad2*) [49] respectively to study localization dependence of CENP-A. Immunostaining with anti-Cse4 (CENP-A) antibodies in depleted levels of each of these subunits of the Dam1 complex revealed that CENP-A localization at the KTs was dramatically reduced as compared to conditions when these proteins were present at wild-type levels (Figure 1A, Figure S1). In order to examine whether CENP-A delocalization is a specific phenomenon associated with depletion of the Dam1 complex, present only in fungal kingdom, we sought to study CENP-A localization upon depletion of the homolog of Nuf2, another evolutionarily conserved outer KT protein, in *C. albicans*. Depletion of Nuf2 in YJB12326 (*MET3*pr*NUF2/nuf2*) showed significantly reduced CENP-A localization as well (Figure 1A). Next, we examined localization of CENP-A in absence of CENP-C/Mif2 in *C. albicans*. CENP-C/Mif2 proteins are evolutionarily conserved inner KT proteins. CENP-A/Cse4 staining in CAMB2 (*PCK1*pr*MIF2/mif2*) grown in repressive media (Glu) for 8 h revealed a significant loss of CENP-A/Cse4 from KTs (Figure 1A). Thus various proteins that are evolutionarily conserved and present at inner, middle and outer KT in many organisms influence CENP-A localization in *C. albicans*.

### The kinetochore super-complex is stabilized by an interdependent coordinated process

Next, to study localization patterns of CENP-C/Mif2 in absence of outer KT proteins, we performed immunostaining using anti-Myc antibodies in J123 (*MET3*pr*DAM1/dam1 MIF2/PCK1*pr*12XMYCMIF2*) and J124 (*MET3*pr*ASK1/ask1 MIF2/2PCK1*pr*12XMYCMIF2*) expressing Myc-tagged CENP-C/Mif2 from the *PCK1* promoter. Similar to CENP-A (discussed above), CENP-C/MycMif2 localization was dramatically reduced when levels of Dam1 or Ask1 were depleted due to growth of J123 or J124 for 8 h under non-permissive conditions (+Cys +Met +Suc) of the *MET3* promoter (Figure 1B). Recently, we demonstrated that in *C. albicans* KT occupancy of these two proteins is dependent on the cellular levels Mis12/Mtw1, an evoutinarily conserved middle KT protein [17]. Thus, assembly of the inner KT is dependent on proteins from middle and outer KT in *C. albicans*.

These results prompted us to further investigate the role of the Dam1 complex on the occupancy of a middle KT protein. KT localized Mtw1-GFP signals that were visible in wild-type conditions were absent when Ask1 or Dam1 was repressed for 8 h in J122 (*MET3*pr*DAM1/dam1 MTW1GFP/MTW1*) or J120 (*MET3*pr*ASK1/ask1 MTW1GFP/MTW1*) (Figure 1C). Together we conclude that integrity of the middle KT is also determined by outer KT proteins. Having established localization dependence of various domains of a KT on each other, we further examined how localization of one protein depends on another at the outer KT. As compared to wild-type, Nuf2-GFP localization at the KT was reduced dramatically when Dam1 was depleted in YJB12289 (*MET3*pr*DAM1/dam1 NUF2GFP/NUF2*) (Figure 1D). These results suggest that integrity of different domains of a KT is interdependent and assembly of various components of a KT is highly coordinated (Figure 1E).

### Integrity of a kinetochore remains unaffected upon disruption of the mitotic spindle

**Figure 2:**
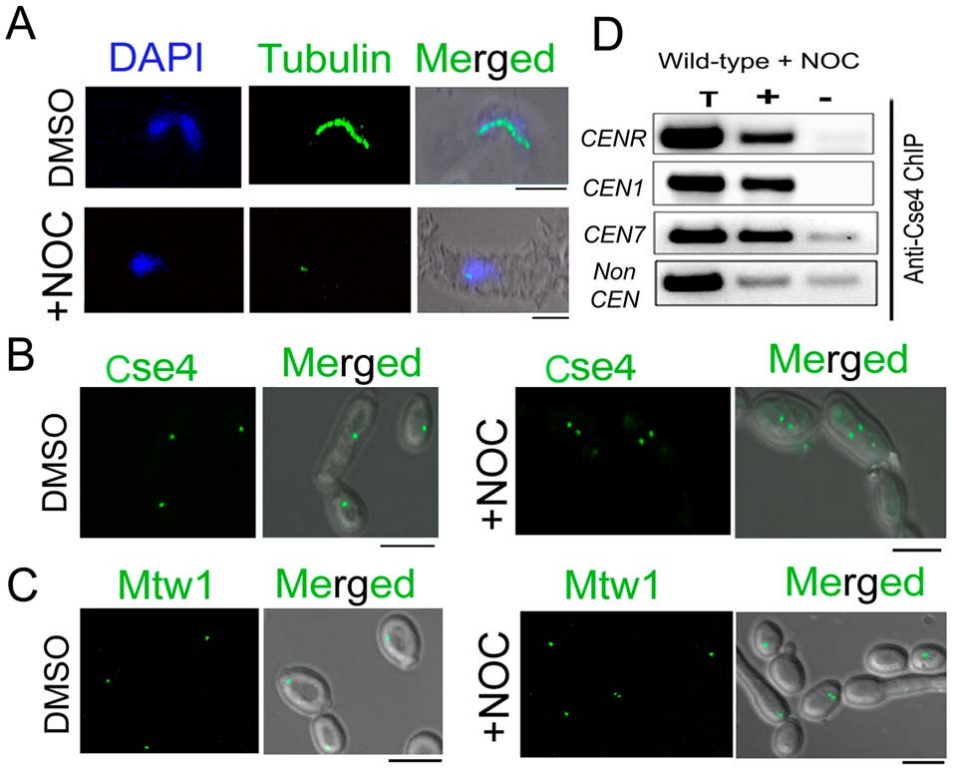
Stability of the kinetochore structure is independent of structural integrity of the mitotic spindle. A) Wild-type BWP17 cells, untreated (DMSO) or treated with nocodazole (+NOC), were fixed and stained with DAPI, and anti-tubulin antibodies. As compared to untreated cells (upper panels), NOC treated cells (lower panels) exhibited a significant loss of tubulin staining indicating a compromised structure of the mitotic spindle as expected by NOC treatment. (B) CENP-A/Cse4-GFP signals were analyzed in absence (DMSO) or presence of NOC (+NOC) in 10118 (*CSE4:GFP:CSE4/cse4*) cells. (C) Similarly, Mtw1-GFP signals were examined in absence (DMSO) or presence of NOC in YJB10695 (*MTW1GFP/MTW1*) cells. No significant change in intensity of either Cse4-GFP or Mtw1-GFP signals in NOC treated cells. (D) PCR analysis on Cse4 ChIP-DNA obtained from NOC treated BWP17 cells. Primers from *CENR* (CaChrR: 1747812–1748023), *CEN1* (CaChr1: 1565723–1565967), *CEN7* (CaChr7: 427369–427560) or 17 kb away from *CEN7* (CaChr7: 444584–444875) (non-*CEN*) were used for PCR analysis. T, total DNA; +, IP DNA with anti-Cse4 antibodies and −, beads only control without antibodies. Bars, 5 µm.

Unlike most organisms including fission yeast and humans, KTs are attached to spindle MTs throughout the cell cycle in *S. cerevisiae* except for a brief period of 2–3 minutes during centromere replication [52], [53]. A similar KT-MT interaction at all stages of the cell cycle is evidenced in *C. albicans* as well [17]. Various proteins from *C. albicans* discussed above have been shown to be essential in KT-MT mediated process of chromosome segregation as severe spindle defects were observed upon depletion of each of these proteins [17], [46], [48], [49]. In this study, we examined whether or not reduced KT localization of various KT proteins was due to impairment of the mitotic spindle caused by depletion of each of these proteins. To test this possibility, we disrupted the mitotic spindle in 10118 (*CSE4:GFP:CSE4/cse4*) expressing GFP-tagged Cse4 by treating cells with a spindle depolymerizing drug nocodazole (NOC). Tubulin staining of these NOC treated cells exhibited disruption of the mitotic spindle structure as expected (Figure 2A). However, no significant change in the intensity of Cse4-GFP signals was observed between NOC treated (mean intensity value = 225±28 a.u.) and untreated (mean intensity value = 227±32 a.u.) cells of 10118 (Figure 2B, Figure S2). A similar experiment to compare Mtw1-GFP levels in NOC treated and untreated cells of YJB10695 (*MTW1*GFP/*MTW1*) exhibited no significant difference (Figure 2C) as well. Localization of Dad2, a subunit of the Dam1 complex, is unaltered in presence or absence of NOC [49]. Together, these results showed that localization of various components of a KT is independent of integrity of the mitotic spindle. CENP-A/Cse4 ChIP analysis further indicated that spindle integrity does not have significant effect on the stability of centromeric chromatin (Figure 2D).

### Partial depletion of several kinetochore proteins leads to disintegration of the clustered kinetochores

**Figure 3:**
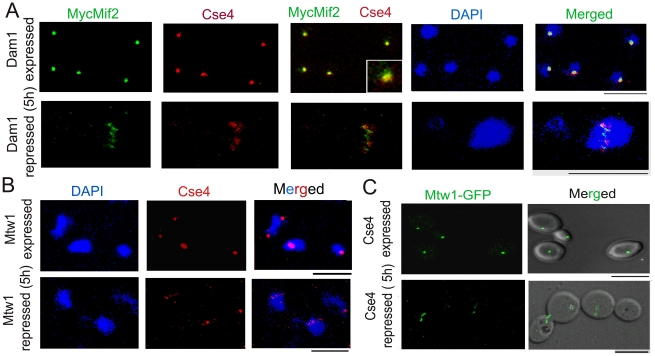
The kinetochore ensemble maintains integrity of clustered kinetochores in *C. albicans*. (A) J123 (*MET3*pr*DAM1/dam1 PCK1*pr*MycMIF2/MIF2*) cells were grown under conditions that expressed (−Cys −Met +Suc) or repressed (+Cys +Met +Suc for 5 h) Dam1. Cells were fixed and stained with DAPI, anti-Cse4 or anti-Myc (Mif2) antibodies. A single cluster of KTs was detected by colocalized CENP-A/Cse4 and CENP-C/Mif2 signals in each cell expressing Dam1. In contrast, declustered KTs, as evidenced by multiple weak Cse4 or MycMif2 signals, were detected in cells with reduced levels of Dam1. (B) CAKS12 (*PCK1*pr*MTW1/mtw1*) cells, grown under conditions that expressed (Suc) or repressed (Glu for 5 h) Mis12/Mtw1, were fixed and stained with DAPI and anti-Cse4 antibodies. Similar KT declustering was visible in Mis12/Mtw1-depleted cells in contrast to clustered KTs in cells expressing this protein. (C) Mis12/Mtw1-GFP signals were observed in YJB11483 (*PCK1*pr*CSE4/cse4 MTW1GFP/MTW1*) cells grown under conditions that expressed (Suc) or repressed (Glu for 5 h) CENP-A/Cse4. Multiple weak Mis12/Mtw1-GFP signals in each cell were observed when CENP-A/Cse4 was repressed. Bars, 5 µm.

In order to understand how absence of a KT protein leads to collapse of an entire KT, we examined the KT structure at reduced (partially depleted) levels of various KT proteins. We observed both CENP-A (Cse4) and CENP-C (MycMif2) signals in wild-type or partially depleted levels of Dam1 or Ask1 in J123 (*MET3*pr*DAM1/dam1 MIF2/PCK1*pr*12XMYCMIF2*) or J124 (*MET3*pr*ASK1)/ask1 MIF2/2PCK1*pr*12XMYCMIF2*). Interestingly, after 4–5 h of Dam1 depletion we observed multiple weak signals of CENP-A (Cse4) and CENP-C (MycMif2) associated with a single nucleus as opposed to a single bright dot-like clustered KTs observed in each wild-type cell (Figure 3A). Ask1-depleted cells showed similar declustered KTs (data not shown). We also observed multiple CENP-A (Cse4) signals in partially depleted Dad2 cells of J108 (*PCK1*pr*DAD2/dad2*) after 4–5 h of growth under non-permissive conditions (Figure S3A). Subsequently, we examined Mtw1-GFP signals by partially depleting Ask1 in J120 (*MET3*pr*ASK1/ask1 MTW1GFP/MTW1*). We observed multiple Mtw1-GFP signals per nucleus in these cells as well (Figure S3B). Nuf2-GFP showed localization patterns in Dam1 depleted conditions (data not shown).

**Figure 4:**
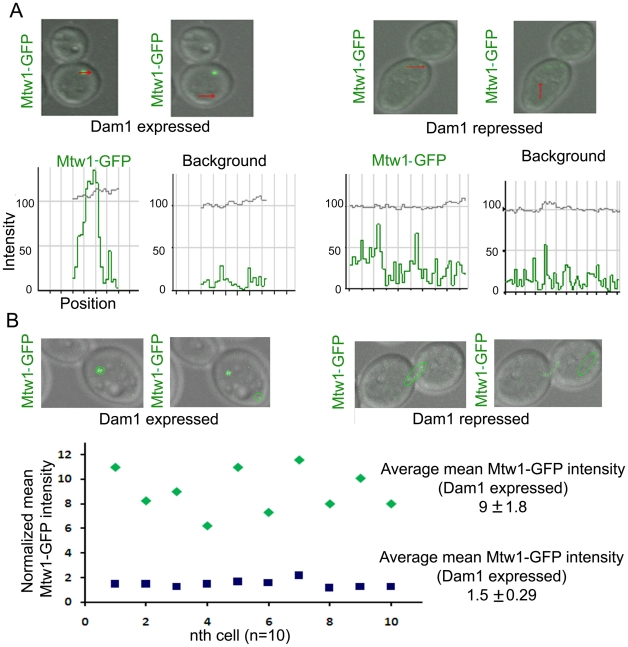
Dam1 depletion results in reduced localization of Mtw1 at the kinetochores. (A) Intensity profiles of Mtw1-GFP or background signals in J122 (*MET3*pr*DAM1/dam1 MTW1GFP/MTW1*) cells having either wild-type or reduced levels of Dam1. (B) Mean intensity values of Mtw1-GFP/KT in wild-type or Dam1 depleted cells were measured using LSM examiner (Carl Zeiss, Germany), normalized with background signals and plotted.

To test whether KT disintegration occurs due to depletion of middle (Mis12/Mtw1) or inner (CENP-A/Cse4) KT proteins as well, we first depleted Mis12/Mtw1 in CAKS12 (*PCK1*pr*MTW1/mtw1*), and examined the integrity of the clustered KTs using anti-Cse4 antibodies. KT disinegration was clearly evident with multiple weak CENP-A signals co-localized with a single nucleus (Figure 3B). Next we monitored the process of Mis12/Mtw1 delocalization upon depletion of CENP-A. YJB11483 (*PCK1*pr*CSE4/cse4 MTW1*GFP/*MTW1*) expressing CENP-A/Cse4 under the *PCK1* promoter was grown either in permissive (+Suc) or non-permissive (+Glu for 5 h) conditions to examine Mtw1-GFP signals (Figure 3C). We observed multiple Mtw1-GFP signals per cell confirming disintegration of the KT cluster in these cells as well (as opposed to wild-type cells where properly integrated KTs remained clustered). Using the LSM examiner analysis tool (Carl Zeiss, Germany), we determined the mean intensity of GFP signals in wild-type clustered KTs and Dam1 depleted declustered KTs (Figure 4). The normalized (against background) average values of mean GFP intensity/KT in wild-type and Dam1 depleted cells were 9±1.8 and 1.5±0.29 respectively. Thus the Mtw1 levels at the disingrated KTs are significantly reduced due to Dam1 depletion. 

### Concerted loss of various kinetochore proteins occurs during the process of kinetochore disassembly

**Figure 5:**
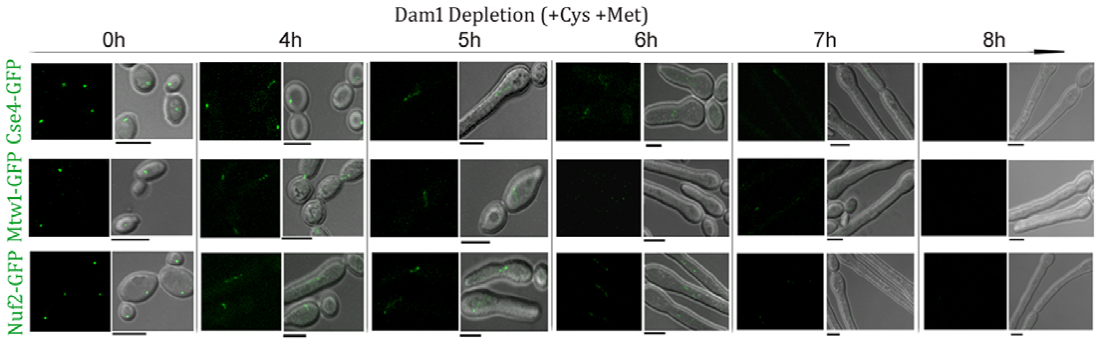
Disintegration of the kinetochore cluster precedes kinetochore collapse. Levels of GFP-tagged inner (Cse4), middle (Mtw1) or outer (Nuf2) KT proteins under gradual repression of Dam1 were monitored at indicated time after shift of strains J127 (*MET3*pr*DAM1/dam1 CSE4:GFP:CSE4/cse4*), J122 (*MET3*pr*DAM1/dam1 MTW1GFP/MTW1*) and YJB12289 (*MET3*pr*DAM1/dam1 NUF2GFP/NUF2*) to non-permissive medium for the *MET3* promoter. In each case GFP (left) and GFP+DIC (right) images were shown. Bars, 5 µm.

Finally, we examined the dynamics of KT disassembly by monitoring the time-dependent localization patterns of several proteins present at various domains of a KT while Dam1 is being gradually depleted. Cse4-GFP, Mtw1-GFP and Nuf2-GFP signals were monitored in J127 (*MET3*pr*DAM1/dam1 CSE4:GFP:CSE/cse4*), J122 (*MET3*pr*DAM1/dam1 MTW1/MTW1GFP*) and YJB12289 (*MET3*pr*DAM1/dam1, NUF2GFP/NUF2*) respectively at various time intervals upon Dam1 depletion (Figure 5). In each case, disintegration of GFP signals from one bright cluster to multiple weakly fluorescent dot-like signals in each cell was observed between 4–5 h of growth in Dam1 repressing media. GFP signals were undetectable after 8 h of Dam1 depletion indicating complete collapse of KT integrity. Similar disintegration dynamics of Mtw1-GFP signals were found between 4–5 h of depletion of other proteins, CENP-A or Ask1, as well (Figure S4). These results indicated a strong correlation between a concerted loss of various domains of a KT and a concomitant reduction in the levels of an essential KT protein.

### Nuclear peripheral localization is maintained even when kinetochores are being disintegrated

**Figure 6:**
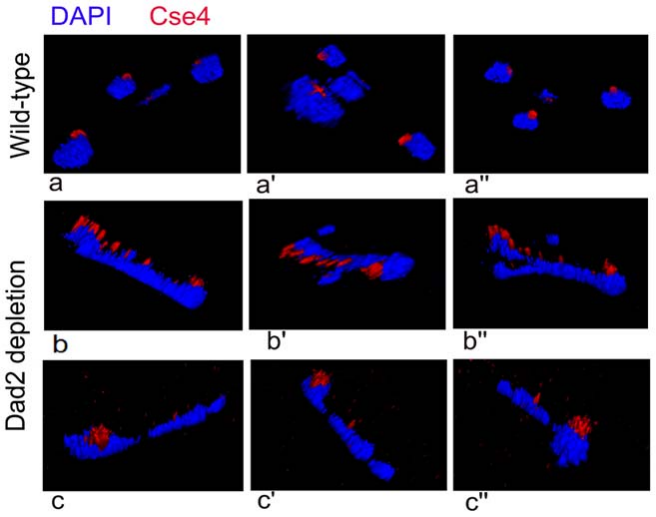
Nuclear peripheral localization is maintained in disintegrated declustered kinetochores. J108 (*PCK1*pr*DAD2*/*dad2*) cells grown for 5 h under non-permissive conditions of the *PCK1* promoter were fixed and stained with DAPI and anti-Cse4 antibodies. Images representing views from different rotational angles (a-a″, b-b′, c-c″) are shown in each case. Each wild-type cell exhibited a single bright dot-like signal of CENP-A/Cse4 at the periphery of the DAPI-stained nucleus (a-a″). Each Dad2-depleted cell, on the other hand, exhibited multiple weak CENP-A/Cse4 signals colocalized with a DAPI stained nucleus (b-b″ and c-c″). Similar to a single intact clustered KTs in the nucleus of each wild-type cell, multiple disintegrated declustered KT signals in the nucleus of each mutant cell were also localized at the nuclear periphery.

All KTs are clustered and such clustered KTs are always localized towards the nuclear periphery (Figure 6, a-a″) suggesting KTs occupy a fixed nuclear territory in wild-type *C. albicans* cells. Interestingly, reconstruction of three dimensional (3D) images revealed declustered KT signals resulting due to Dad2 depletion in J108 (*PCK1*pr*DAD2/dad2*) retained nuclear peripheral localization. Since peripheral nuclear localization of declustered KTs is maintained (Figure 6, b-b″, c-c″), we speculate that KTs, whether clustered or not, remain largely attached to nuclear periphery through some components that are not affected due to depletion of an individual KT protein.

### An intact kinetochore stabilizes centromeric chromatin

**Figure 7:**
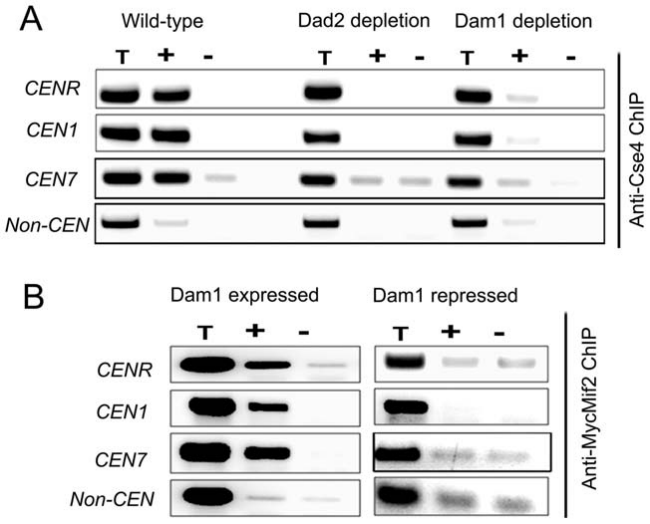
The Dam1 complex maintains inner kinetochore assembly including integrity of centromeric chromatin. (A) PCR analysis on Cse4 ChIP DNA obtained from wild-type BWP17, Dam1-depleted J102 (*MET3*pr*DAM1/dam1*) or Dad2-depleted J108 (*PCK1*pr*DAD2*/*dad2*) cells grown for 8 h in non-permissive conditions. Primers from *CENR* (CaChrR: 1747812–1748023), *CEN1* (CaChr1: 1565723–1565967), *CEN7* (CaChr7: 427369–427560) or 17 kb away from *CEN7* (CaChr7: 444584–444875) (non-*CEN*) were used for PCR analysis. T, total DNA; +, IP DNA with anti-Cse4 antibodies and −, beads only control without antibodies. CENP-A/Cse4 recruitment was found to be highly reduced at centromeres in Dam1- or Dad2-depleted cells as compared to wild-type. (B) MycMif2 ChIP-PCR analysis with DNA obtained from J123 (*MET3*pr*DAM1/dam1 PCK1*pr*MycMIF2/MIF2*) either expressing Dam1 (WT) or depleted of it. Same primer pairs from *CENR*, *CEN1*, *CEN7* or non-centromeric region *LEU2* (non *CEN*) were used for PCR analysis. T, total DNA; +, IP DNA with anti-Myc antibodies and −, beads only control without antibodies.

To examine the status of centromeric chromatin when KT integrity is lost, ChIP assays with anti-Cse4 antibodies were performed in BWP17 (wild-type), J102 (*MET3*pr*DAM1/dam1*) and J108 (*PCK1*pr*DAD2/dad2*) strains grown in non-permissive media for 8 h. These experiments revealed a drastic decrease in CENP-A binding to *CEN*s in Dam1 or Dad2 depleted cells as compared to wild-type cells confirming that integrity of CENP-A-bound centromeric chromatin is highly affected in these mutants (Figure 7A). Since the localization of CENP-C/Mif2 was also affected when Dam1 was depleted we tested centromere occupancy of CENP-C/Mif2 in J125 (*MIF2*/*PCK1*pr*12XMYCMIF2*) or depleted levels of Dam1 in J123 (*MET3*pr*DAM1)/dam1 MIF2/PCK1*pr*12XMYCMIF2*) by ChIP assays with anti-MycMif2 antibodies (Figure 7B). The ChIP-PCR analysis confirmed that CENP-C/Mif2 occupancy at centromeres was also dramatically reduced in Dam1 depleted cells as compared to wild-type cells. We have recently shown that, Mtw1/Mis12 is required for inner kinetochore assembly including localization of CENP-A and CENP-C [17]. Together these results confirmed that Dam1 is required for integrity of centromeric chromatin in *C. albicans*.

### CENP-A is unstable in absence of an essential KT protein

**Figure 8:**
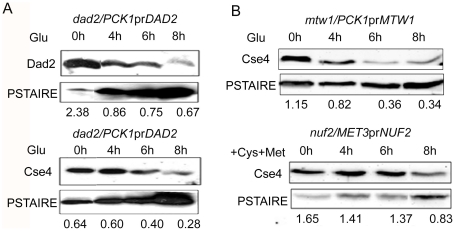
CENP-A is protected by the kinetochore ensemble. (A) J108 (*PCK1*pr*DAD2*/*dad2*) was grown overnight in media expressing Dad2 (+Suc), transferred to media that repressed Dad2 (+Glu), and cells were harvested at specific time intervals as shown. Western blot analysis was performed using anti-Dad2, anti-Cse4 or anti-PSTAIRE antibodies with cell lysates prepared from these cells. Both Dad2 (left panels) and CENP-A/Cse4 (right panels) protein levels showed a gradual decrease as time of repression of Dad2 prolonged. PSTAIRE was used as the loading control. Increasing amount of protein was loaded to visualize the reduced Dad2 or CENP-A/Cse4 protein signals in these western blots. (B) Western blot analysis using anti-Cse4 and anti-PSTAIRE antibodies with cell lysates prepared from conditional mutant strains CAKS12 (*PCK1*pr*MTW1/mtw1*) and YJB12326 (*MET3*pr*NUF2/nuf2*) before (0 h) and at indicated time of incubation in non-permissive media after shift. Relative levels of Cse4 or Dad2 normalized by corresponding levels of PSTAIRE are shown.

Since centromeric chromatin is disintegrated when various KT proteins are depleted, we examined protein levels of CENP-A in these conditions to find out the fate of CENP-A molecules that are no longer associated with centromeres. We prepared lysates from J108 (*PCK1*pr*DAD2*/*dad2*) grown overnight in Suc (expressed condition) or various time intervals after transferring in Glu (repressed condition), and performed western blot analysis to measure the levels of both Dad2 and CENP-A. A decrease in Dad2 protein levels in cells grown at increasing time in Glu confirmed time-dependent repression of Dad2 expression by the *PCK1* promoter (Figure 8A, top panel). Strikingly, a concomitant reduction in CENP-A levels, as determined by western blot analysis using anti-Cse4 antibodies, with decreasing levels of Dad2 was also observed (Figure 8A, bottom panel). To examine the fate of CENP-A in reduced levels of other subunits of the Dam1 complex, total cell lysates were prepared from strains where Dam1 (J102), Ask1 (J104), or Spc19 (J106) was either expressed or repressed for 8 h. As observed in Dad2-depleted cells, CENP-A levels in the total cell lysate were found to be highly reduced in absence of each of these proteins (Figure S5). Next we examined CENP-A stability in depleted conditions of CENP-C/Mif2, Mis12/Mtw1, and Nuf2 - evolutionarily conserved inner, middle and outer KT proteins respectively. Total cell lysate was prepared from each sample collected at different time points during Mif2/CENP-C, Mis12/Mtw1 or Nuf2 reprefssion in CAMB2 (*PCK1*pr*MIF/mif2*), CAKS12 (*PCK1*pr*CSE4/cse4*) or YJB12326 (*MET3*pr*NUF2/nuf2*). Western blot analysis of these cell lysates with anti-Cse4 antibodies confirmed a decrease in CENP-A levels when either CENP-C (Figure S5), Mis12 or Nuf2 (Figure 8B) was depleted. Thus, CENP-A protein stability is dependent on wild-type levels of several KT proteins.

### The proteasomal mediated pathway is involved in CENP-A degradation in absence of Dam1

**Figure 9:**
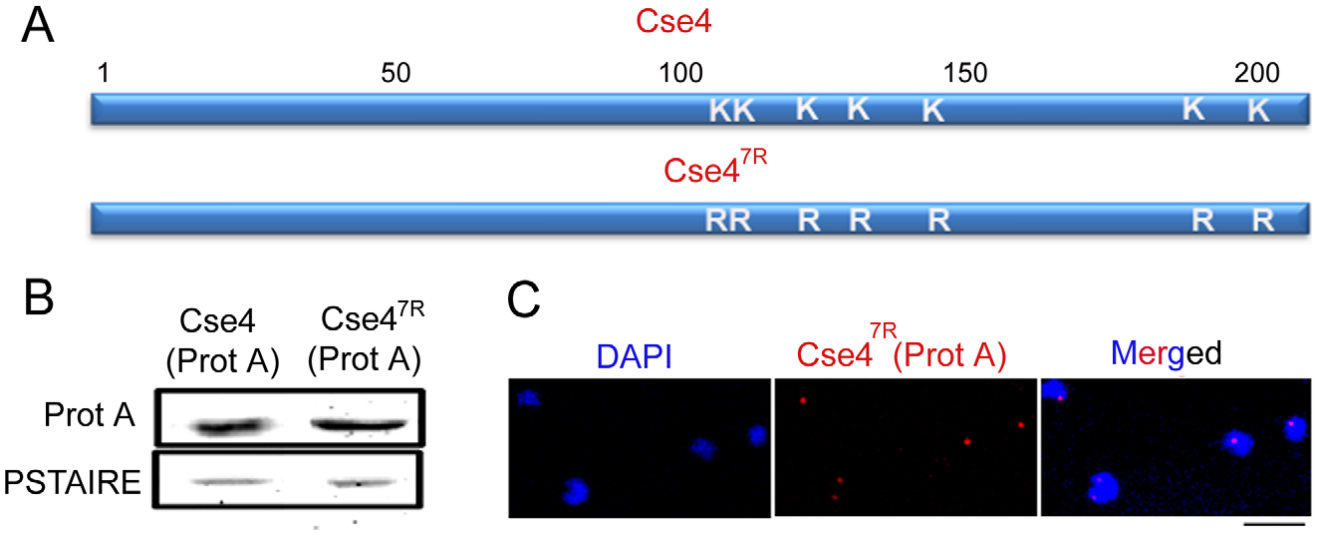
Cse4^7R^ -Prot A is functional in *C. albicans*. (A) Each of the 7 lysine (K) residues of Cse4 ORF shown in schematic has been mutated to arginine (R) residues by site-directed mutagenesis. (B) Lysates from strains expressing wild-type J128 (*CSE4-*Prot A/*cse4*) and J129 (*CSE4^7R^*-Prot A/*cse4*) Cse4-Prot-A or Cse4^7R^–Prot-A were subjected to western blot analysis with anti-Prot-A or anti-PSTAIRE antibodies. (C) Indirect immunolocalization by anti-Prot A (Cse4) antibodies in J129 (*CSE4^7R^*-Prot A/*cse4*) exhibited normal KT localization patterns of Cse4^7R^. Bar, 5 µm.

To investigate whether increased levels of CENP-A could rescue KT integrity in depleted levels of a KT protein we sought to express a mutant stable form of CENP-A in *C. albicans*. In budding yeast *S. cerevisiae* Cse4/CENP-A at non-centromeric regions is degraded by the proteasomal mediated degradation pathway which is partially prevented by replacement of all lysine residues with arginine residues in ScCse4 ORF. Using site-directed mutagenesis, all seven lysine residues in Cse4 ORF in *C. albicans* strain (CAKS2b) were replaced by arginine residues to construct the strain J129 (*CSE4^7R^-*Prot A/*cse4*) where Protein A (Prot A) tagged mutated Cse4^7R^ is the only source of CENP-A (Figure 9A). All the changes were confirmed by DNA sequencing (Figure S6). Western blot analysis (Figure 9B) and immunolocalization (Figure 9C) using anti-Prot A antibodies revealed that Cse4^7R^ -Prot A is functionally expressed in *C. albicans*.

**Figure 10:**
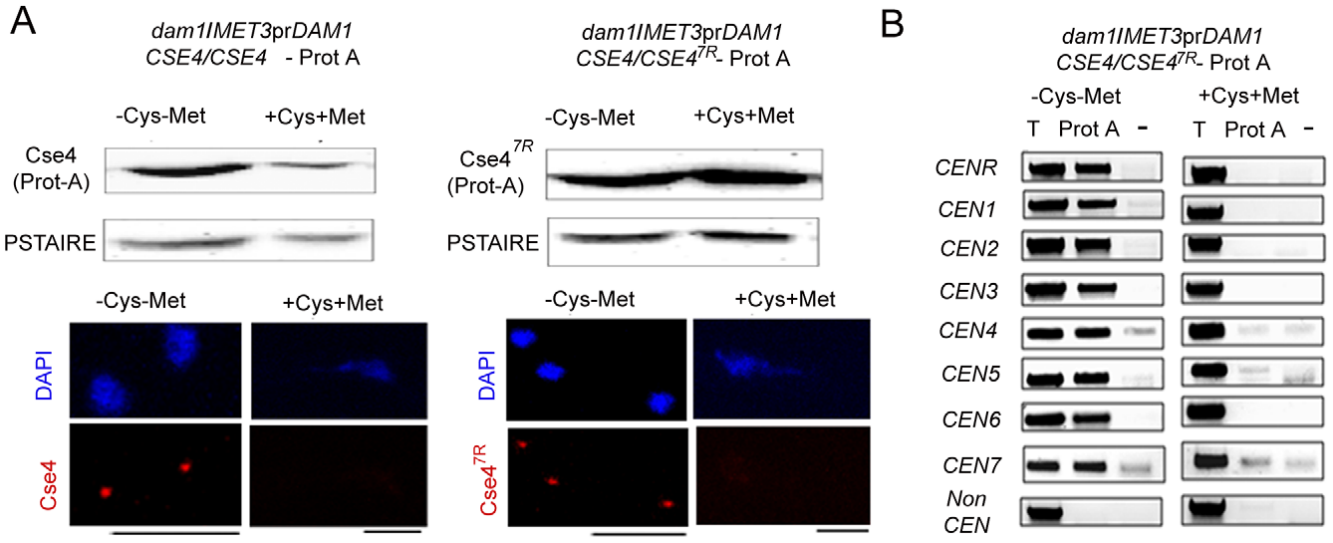
Depletion of Dam1 leads to degradation of CENP-A/Cse4 through the ubiquitin-mediated proteasomal degradation pathway. (A) Lysates were prepared from strains J130 (*MET3*pr*DAM1)/dam1 CSE4*-Prot A/*CSE4*) and J131 (*MET3*pr*DAM1/dam1 CSE4^7R^*-Prot A/*CSE4*) expressing wild-type Cse4 -Prot A (left) or mutant Cse4^7R^ -Prot A (right) grown under conditions where Dam1 is either expressed (−Cys −Met) or repressed (+Cys +Met). Western blot analysis was performed with these lysates using anti-Prot A or anti-PSTAIRE antibodies. Indirect immunolocalization with anti-Cse4 antibodies revealed that recruitment of either wild-type Cse4 or mutant Cse4^7R^ at the kinetochore is compromised in absence of Dam1. (B) Prot A (Cse4) ChIP assays in strain J131 (*MET3*pr*DAM1/dam1 CSE4^7R^*-Prot A/*CSE4*) confirmed that stable mutated Cse4^7R^ is unable to maintain centromeric chromatin in absence of Dam1. *CEN*, centromeres; T, Total input DNA, Prot-A, immunoprecipitated DNA with anti-Prot A antibodies, −, beads only control. Bars, 5 µm.

To examine the effect of Dam1 depletion on the stability of Cse4^7R^, we expressed Cse4-Prot A and Cse4^7R^-Prot A in the Dam1 conditional mutant. Western blot analysis in J130 (*MET3*pr*DAM1)/dam1 CSE4*-Prot A*/CSE4*) or J131 (*MET3*pr*DAM1/dam1 CSE4^7R^-*Prot A/*CSE4*) revealed that while wild-type Cse4-Prot A was degraded, Cse4^7R^ -Prot A remained stable upon Dam1 depletion (Figure 10A). This suggests that the proteasomal mediated pathway is involved in degradation of unbound CENP-A in *C. albicans*.

### Cse4^7R^ fails to rescue kinetochore disassembly triggered by depleted levels of Dam1

To test whether increased levels of CENP-A (Cse4^7R^ ) could rescue KT disintegration caused by depletion of Dam1, we analysed Cse4^7R^ localization at the KTs in Dam1 mutant. Immunolocalization using anti-Cse4 antibodies in J130 (*MET3*pr*DAM1)/dam1 CSE4-*Prot A*/CSE4*) or J131 (*MET3*pr*DAM1/dam1 CSE4^7R^-*Prot A/*CSE4*) cells revealed that similar to wild-type Cse4, Cse4^7R^ failed to localize at the KTs in absence of Dam1 (Figure 10A). Prot A ChIP analysis from J131 (*MET3*pr*DAM1/dam1 CSE4^7R^*-ProtA/*CSE4*) cells showed a significant drop in Cse4^7R^ binding to centromeres of all chromosomes under Dam1 depleted conditions. Thus Dam1 depletion leads to disintegration of centromeric chromatin containing wild-type CENP-A (Cse4) and stable form of CENP-A (Cse4^7R^ ) in the same manner (Figure 10B).

### Newly synthesized CENP-A fails to localize at the kinetochores in absence of Dam1

**Figure 11:**
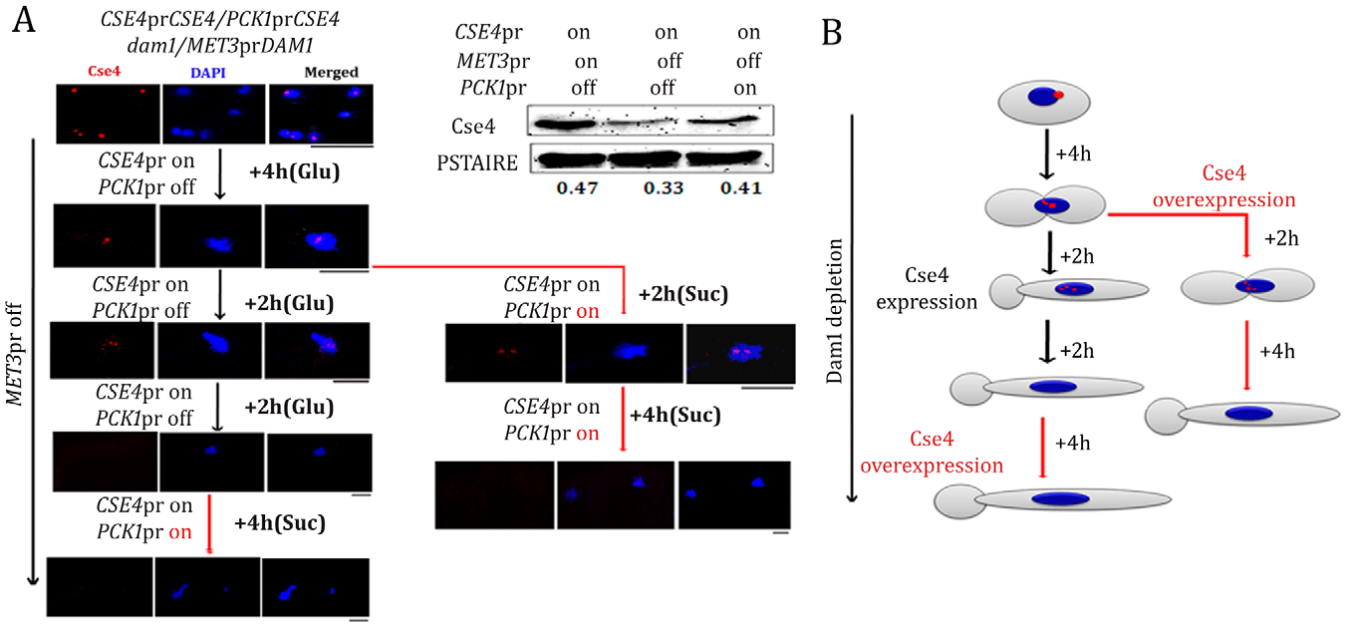
Kinetochore localization of newly synthesized CENP-A/Cse4 is compromised in absence of wild-type levels of Dam1. (A) Strain YJB11990 (*PCK1*pr*CSE4/CSE4, MET3*pr*DAM1/dam1*) was grown in conditions where CENP-A/Cse4 is expressed from its native promoter and Dam1 is expressed from the *MET3* promoter (−Cys −Met +Glu). Overnight grown cells were then transferred to a media (+Cys +Met +Glu) that represses Dam1 expression but allows Cse4 expression only from its native promoter. After 4 h of growth in such conditions, one fraction (Fraction-I) of the culture was grown in the same media (+Cys +Met +Glu) for an additional 4 h. The second fraction (Fraction II) of the culture was transferred to a condition that overexpressed Cse4 but kept Dam1 repressed (+Cys +Met +Suc) for additional 6 h. Subsequently, Fraction-I grown for 8 h in +Cys +Met +Glu containing media was transferred to +Cys +Met +Suc and grown for an additional 4 h. Samples were collected from various time points indicated, fixed and stained with DAPI and anti-Cse4 antibodies. (B) Schematic showing the fate of newly synthesized CENP-A/Cse4 in Dam1 depleted conditions. Bars, 5 µm.

We next examined whether newly synthesized CENP-A molecules can be recruited to the KT once the process of KT disassembly is initiated under Dam1 depleted conditions. We utilized YJB11990 (*PCK1*pr*CSE4/CSE4 MET3*pr*DAM1/dam1*) in which *CSE4* and *DAM1* are placed under control of the *PCK1* and *MET3* promoters respectively. We studied the fate of newly synthesized CENP-A molecules by inducing expression of CENP-A/Cse4 (+Suc) while Dam1 is kept in repressed state (+Cys +Met). To achieve this, YJB11990 was grown in Dam1 repressible conditions till the point where (a) KTs starts to decluster (4 h) or (b) KTs are disintegrated (8 h) (Figure 11A). These cells were then transferred to media that represses the *MET3* promoter (+Cys +Met to keep Dam1 depleted) but induces the *PCK1* promoter (+Suc to overexpress Cse4) and grown for an additional 4 h. Expression of new CENP-A/Cse4 molecules in such conditions was confirmed by western blot analysis (Figure 11A). Indirect immunolocalization using anti-Cse4 antibodies revealed that newly synthesized CENP-A/Cse4 molecules expressed from the induced *PCK1* promoter (Figure 11A) could not be recruited at KTs under Dam1 repressible conditions (Figure 11B).

## Discussion

The kinetochore is a complex DNA-protein structure where more than 80–100 proteins assemble [23], [54], [55]. Although genetic, biochemical and microscopy studies on localization dependence of several key evolutionarily conserved KT proteins suggest a possible hierarchical assembly of a three-layered KT structure, it has also been proposed that the KT may not be assembled in a single linear order. Bloom and colleagues indicated *CEN* DNA bending caused by the CBF3 complex in *S. cerevisiae* or by CENP-B binding to alpha-satellite *CEN* DNA in mammalian centromeres may provide proper geometry for KT formation, a process that may be evolutionarily conserved [33]. In this work, we chose to delineate the process of KT assembly in another budding yeast *C. albicans*. *De novo* centromere formation does not take place in *C. albicans* suggesting that centromere formation is epigenetically determined [45]. It has also been shown that centromeres can form very efficiently on non-centromeric locations in *C. albicans* when a native centromere is deleted from a chromosome [37]. All these observations point toward sequence independent assembly of a KT. *C. albicans* lacks homologs of sequence-specific DNA binding centromeric proteins such as subunits of the point centromere-specific CBF3 complex or regional centromere-specific CENP-B. Neocentromere formation does not take place in *S. cerevisiae* as centromere identity is strictly maintained in sequence-dependent manner by sequence-specific DNA binding proteins. CENP-B is absent from the KT assembled on human neocentromeres unlike most of other KT proteins [38] suggesting that the mechanism of KT assembly on neocentromere might be different from that of an endogenous centromere in humans. In absence of any known KT protein that binds to a specific sequence motif as well as absence of any conserved DNA sequence common to all *CEN*s, *C. albicans* provides a unique system to study KT assembly that relies on an epigenetic sequence-independent process.

### CENP-A localization depends on various kinetochore proteins in *C. albicans*

CENP-A is required for localization of many KT proteins [12], [14], [15]. In *S. cerevisiae*, inner and middle KT proteins (Ndc10, Mif2, Mtw1, and Okp1) showed 50% decrease in occupancy at the active conditional centromere (*cCEN*) in absence of CENP-A/Cse4, whereas the middle (Ctf19) and many outer (Ndc80, Dam1, Ask1, and Stu2) KT proteins completely failed to localize [43]. This suggests that CENP-A/Cse4 is the initiator of KT assembly. However, several kinetochore proteins do not require CENP-A for localization, suggesting that CENP-A independent species-specific KT assembly pathways also exist in certain organisms [15], [18], [56]. CENP-A localization has been shown to influence Mis12 localization in *S. cerevisiae*, *D. melanogaster* and humans but not in *S. pombe* [55], [57]–[59]. On the other hand, Mis12 does not influence localization of CENP-A in most organisms except in *C. albicans* where CENP-A and Mis12 localization is interdependent [15], [55], [58], [59]. Depletion of CENP-A affects CENP-C localization in *S. cerevisiae*, *C. elegans* and humans but CENP-C has no effect on CENP-A localization in these organisms [13], [14].

In this study, we examined how CENP-A localization is influenced by other KT proteins in *C. albicans*. Intriguingly, centromere localization of CENP-A was dramatically reduced due to depletion of inner (Mif2/CENP-C) or outer (Dam1 complex and Nuf2) KT proteins. Recently, we showed that a middle KT protein Mis12/Mtw1 homolog *C. albicans* influences assembly of two inner KT proteins CENP-A and CENP-C [17]. Thus localization dependence of CENP-A on various KT proteins varies from species to species.

### An interdependent coordinated protein circuitry stabilizes the kinetochore structure

Since it is unusual and striking that outer KT proteins influence localization of CENP-A, we further investigated localization dependence of various other proteins to determine the sequence of events that lead to KT formation on unique short regional centromeres of *C. albicans*. An unprecedented observation of collapse of the KT architecture in absence of an essential protein from a KT in *C. albicans* raises an important question. How do KTs assemble in *C. albicans*? KT disassembly due to depletion of various proteins suggests that KT assembly is probably not a step-wise process in *C. albicans*. We propose that KT proteins of various sub-complexes assemble in a unique interdependent concerted manner to form and stabilize the macromolecular KT architecture in *C. albicans* Our results thus indicate that the KT in *C. albicans* may not even have a layered structure, unlike the one observed in humans.

### Stability of the kinetochore structure is independent of structural integrity of the mitotic spindle

Since all proteins we analyzed in this study have been previously shown to be essential for the proper dynamics of a mitotic spindle in *C. albicans* [17], [48], [49] we investigated whether or not an intact mitotic spindle is required for maintaining integrity of KTs. Localization of CENP-A or Mis12 was found to be unaffected in presence of a MT-depolymerizing drug nocodazole. In NOC treated cells tubulin staining showed two weak dot-like signals probably representing SPBs after duplication. Cse4 and Mtw1 GFP signals in NOC treated cells are also seen as two dots situated close to each other. Thus we conclude that stabilization of the KT structure is independent of structural integrity of the mitotic spindle.

### The kinetochore ensemble maintains integrity of the kinetochore cluster in *C. albicans*

Like *S. cerevisiae*, KT-MT interaction is established early during S phase in *C. albicans*. Live cell imaging by time-lapse microscopy in our previous study revealed that all centromeres are clustered together throughout the cell cycle in *C. albicans* [17]. Moreover, similar localization patterns of these clustered centromeres at the nuclear periphery were observed both in *S. cerevisiae* and *C. albicans*. In absence of a metaphase plate in budding yeasts [60], clustered centromeres may provide a platform for MT attachment and synchronous separation of sister chromatids during anaphase. Conditional KT mutants provided us an opportunity to follow the process of KT disassembly in a time dependent manner during gradual depletion of various KT proteins in *C. albicans*. Our results clearly indicate that disintegration of the KT cluster is a common intermediate step before KT collapse and each component is required to keep all the KTs clustered in *C. albicans*. Chromosome confirmation capture (3C) assay to study interaction among centromeres of different chromosomes in *S. cerevisiae* revealed that crosslinking frequencies between different centromeres is significantly higher than other chromosomal sites except telomeric regions [61]. A recent study, where chromosome confirmation capture on chip (4C) and massively parallel sequencing were used to globally capture inter and intrachromosomal interactions in *S. cerevisiae*, demonstrates centromeres as the chromosome landmarks that mediate interchromosomal interaction [32]. The clustering of centromeres that marked the primary point of engagement between different chromosomes was the most striking feature of the inter-chromosomal contacts. In the light of these observations in *S. cerevisiae* and our results in this study, we speculate that interchromosomal interaction at the centromeres may facilitate stabilization of the kinetochore ensemble as depletion of various KT proteins leads to disintegration of clustered centromeres in both species.

### An intact kinetochore protects CENP-A from degradation

ChIP analysis and immunolocalization studies confirmed that CENP-A occupancy is severely impaired when an essential KT protein is depleted from *C. albicans* cells. We predicted that disintegration of centromeric chromatin in absence of KT proteins may expose free CENP-A molecules for cellular degradation. Indeed, western blot analysis confirmed that cellular CENP-A protein levels were drastically reduced in KT mutants tested. Since integrity of centromeric chromatin is also dependent upon individual KT proteins which in turn help in maintaining overall KT integrity, it is tempting to speculate that establishment of centromeric chromatin and assembly of KTs start together in a coordinated way in *C. albicans*.

### The proteasomal mediated pathway of CENP-A degradation is evolutionarily conserved

The ubiquitin mediated degradation pathway is one of the major pathways that degrade CENP-A/Cse4 in *S. cerevisiae* and CENP-A/CID in *Drosophila*. In Dam1 depleted strain where wild-type Cse4 is unstable, the mutant Cse4^7R^ showed higher stability suggesting a similar CENP-A degradation pathway is active in *C. albicans*. Thus, although the process of CENP-A recruitment on point and regional centromeres may differ but the mechanism that regulates CENP-A stability to prevent ectopic KT formation (especially in *C. albicans* where efficiency of neocentromere formation is remarkably high) seem to be conserved among organisms carrying point and regional centromeres.

### Dam1 is required for CENP-A recruitment at the kinetochores

Wild-type CENP-A is unstable when the KT ensemble is disintegrated and centromeric chromatin is disrupted in absence of an essential KT protein. It is possible that reduction in CENP-A levels is the cause not the consequence of KT disassembly under such conditions. We wondered whether expression of a stable form of CENP-A (Cse4^7R^) can prevent disintegration of the KT structure in absence of Dam1. We examined stable mutant CENP-A, Cse4^7R^ localization at the KT in Dam1 depleted cells and found that increasing the stability of CENP-A (confirmed by western blot analysis) does not prevent CENP-A delocalization at the KT in absence of Dam1. The Cse4^7R^ failed to maintain integrity of centromeric chromatin as well. Next we sought to provide new CENP-A molecules using an inducible promoter (*PCK1*) to examine if newly synthesized CENP-A can be recruited to the KTs in absence of Dam1. However, these newly synthesized CENP-A molecules (detected by western blot analysis) failed to rescue KT integrity further confirming that an individual KT protein is absolutely essential for protecting the centromeric bound CENP-A by maintaining the integrity of the KT ensemble that is laid on the foundation of CENP-A associated centromeric chromatin.

**Figure 12:**
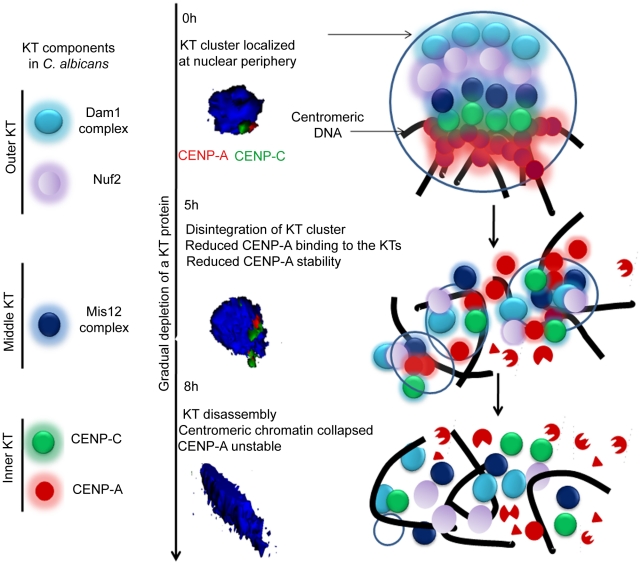
The dynamics of kinetochore assembly/disassembly in *C. albicans*. Cellular levels of an essential KT protein maintain centromere clustering and integrity of a KT formed on the foundation of CENP-A containing centromeric chromatin. The clustered KTs maintain integrity of centromeric chromatin to protect CENP-A from degradation. A gradual depletion (5 h) of any of the proteins from the inner, middle or outer KT results in declustering of KTs followed by disassembly (8 h) of the KT ensemble. Disassembled KTs fail to protect centromeric chromatin. CENP-A molecules that are no longer assembled into centromeric chromatin eventually get degraded by the proteasomal mediated pathway. The 3D images represented were constructed from original images in Figure 3.

Figure 12 shows a possible pathway of KT destabilization due to depletion of an essential KT protein in *C. albicans*.

## Materials and Methods

### Strain construction

Strains and primers used in this study are listed in Table S1 and Table S2 respectively.

#### Construction of conditional mutants expressing epitope tagged kinetochore proteins

To visualize Mtw1 under Ask1 and Dam1 depleted conditions, we constructed Dam1 and Ask1 conditional mutant strains in YJB10695 (*MTW1/MTW1GFP*). The first copy of each of *DAM1* or *ASK1* was replaced by *HIS1* in YJB10695. We used long primers (100–120 bp) (Table S2) whose 5′ end were homologous to sequences upstream and downstream of each gene, and 3′ ends were homologous to *HIS1* gene. Using these primers deletion cassettes for *ASK1* and *DAM1* were amplified from the plasmid HIS1GFP [62]. Each deletion construct which carries sequences upstream and downstream of *ASK1* or *HIS1* flanked by *HIS1* gene was used to transform YJB10695. Transformants were then selected on complete media lacking histidine (CM-His). In resulting strains J119 or J121 the remaining wild-type allele of *DAM1* or *ASK1* was placed under control of the *MET3* promoter [50]. We replaced *CaURA3* with *C. dubliniensis ARG4* in the plasmids pAsk1MET3 or pDam1MET3 [49] that contained part of the corresponding ORF including the start codon of *ASK1* or *DAM1* respectively next to the *MET3* promoter. Each of these resulting plasmids was linearized with *Cla*I to transform J119 or J121 and transformants were selected on media lacking arginine (CM-Arg). Resulting conditional Ask1 and Dam1 mutant strains were named J120 and J122 respectively.

To visualize Mif2 signals in Ask1 or Dam1 depleted conditions, we constructed strains expressing Myc-tagged Mif2 in Dam1 or Ask1 conditional mutant strains. A cassette containing *PCK1*pr*MycMIF2* was amplified from strain CAMB2 [46] using primers Mif2PckMycPst1-F and Mif2pck1SacII-R and subsequently cloned into *Pst*I and *Sac*II sites of pSF2A vector [63] that contained the nourseothricin (NAT) marker. The resulting plasmid was linearized using *Hpa*I and introduced into J102 (Dam1 conditional mutant) or J104 (Ask1 conditional mutant) strains to get J123 (*dam1/MET3DAM1*, *MIF2/PCK1*pr*MycMIF2*) or J124 (*ask1/MET3ASK1*, *MIF2/PCK1*pr*MycMIF2*).

#### Construction of strains expressing Cse4-Prot A and Cse4^7R^-Prot A

The *CSE4* ORF was cloned into *Sac*II and *Spe*I sites of pBluescript to construct pBSCSE4. All seven lysine residues were changed into arginine residues in the plasmid pBSCSE4 using a site-directed mutagenesis kit (Stratagene, Cat # 200522) to construct pBSCSE4^7R^. The TAP cassette (containing *URA3* and TAP that contains Prot A) and *CSE4* downstream sequences were cloned as *Nco*I/*Kpn*I and *Kpn*I/*Sac*I fragments respectively into the corresponding sites of pUC19 to construct pCSE4DSTAP. The *CSE4* ^7R^ fragment was amplified from pBSCSE4^7R^, digested with *Sal*I and *Nco*I and cloned into corresponding sites of pCSE4DSTAP to yield pCSE4^7R^TAP. Similarly the *CSE4* fragment was cloned into *Sal*I and *Nco*I sites of pCSE4DSTAP to construct pCSE4TAP. Both pCSE4TAP and pCSE4^7R^TAP were linearized using *Xho*I and these linearized fragments were used to transform CAKS2b (*CSE4/ cse4::hisG* ) to give rise to J128 (*CSE4-TAP(URA3)/cse4::hisG* ) and J129 (*CSE4^7R^-TAP(URA3)/cse4::hisG)* respectively. Subsequently, J126 (*dam1::HIS1 MET3*pr*DAM1(ARG4)/dam1::HIS1)* was transformed with *Xho*I-digested pCSE4TAP and pCSE4^7R^TAP separately to give rise to J130 (*dam1::HIS1 MET3*pr*DAM1(ARG4)/dam1::HIS1 CSE4-TAP(URA3)/CSE4)* and J131 (*dam1::HIS1 MET3*pr*DAM1(ARG4)/dam1::HIS1 CSE4^7R^-TAP(URA3)/CSE4)* respectively.

### Media and growth conditions

Conditional mutant strains of Dam1 (J102, J121, J122, J127, J130, J131, YJB11990 and YJB12289), Ask1 (J104, J120, and J124), Spc19 (J106) and Nuf2 (YJB12326) that carry *DAM1*, *ASK1 SPC19* and *NUF2* respectively under control of the *MET3* promoter were grown in YPDU (1% yeast extract, 2% peptone, 3% glucose and 0.01% uridine) as permissive media and YPDU+5 mM cysteine (+Cys)+5 mM methionine (+Met) as non-permissive media. Conditional mutant strains of Mif2 (CAMB2, J123, J124 and J125), Dad2 (J108), Cse4 (CAKS3b and YJB11483) and Mtw1 (CAKS12) that carry *MIF2*, *DAD2*, *CSE4* and *MTW1* respectively under control of the *PCK1* promoter were grown in YPSU (1% Yeast Extract, 2% Peptone, 2% Succinate and 0.01% Uridine) as permissive media and YPDU as non-permissive media. All *C. albicans* strains were grown at 30°C.

### GFP imaging

*C. albicans* conditional mutants with GFP tagged KT proteins grown overnight in inducing media were transferred to repressible media at initial OD_600_ - 0.150. Cells were harvested at various time intervals after growth in repressible media. Harvested cells were resuspeneded in 50% glycerol and subsequently imaged using a confocal microscope (Zeiss LSM 510 META). Images were further processed by Adobe Photoshop.

### Nocodazole treatment

For nocodazole treatment, cells were grown overnight in YPDU, reinoculated in YPDU with an initial OD_600_ = 0.2. Nocodazole (Sigma, Cat # M1404) was added at a concentration of 20 µg/ml when OD_600_ = 0.4 (1 generation) was achieved. Cells were grown for an additional 4 h before harvesting for immunolocalization and ChIP assays.

### Chromatin immunoprecipitation (ChIP) assay

ChIP assays were performed using a protocol described previously [49]. An exponentially growing culture of *C. albicans* strain was fixed with 1% formaldehyde for 15 min. The reaction was quenched for 5 min at room temperature using glycine to a final concentration of 125 mM. Cells were washed and suspended in resuspension buffer (0.2 mM Tris-HCl pH, 9.4, 10 mM DTT). Resuspended cells were incubated at 30°C for 15 min on a shaker at 180 rpm. Cells were washed and resuspended in spheroplasting buffer (1.2 M Sorbitol, 20 mM Na-HEPES, pH 7.5). Spheroplasting (95%) was performed using lyticase (Sigma, Cat # L2524) at 30°C at low speed. Spheroplasting was stopped by adding ice-cold postspheroplasting buffer (1.2 M Sorbitol, 1 mM MgCl_2_, 20 mM Na-PIPES, pH 6.8). Spheroplasts were subsequently washed with ice-cold 1× PBS, Buffer I (0.25% TritonX-100, 10 mM EDTA, 0.5 mM EGTA, 10 mM Na-HEPES, pH 6.5), Buffer II (200 mM NaCl, 1 mM EDTA, 0.5 mM EGTA, 10 mM Na-HEPES) and finally resuspended in extraction buffer (140 mM NaCl, 1 mM EDTA, 50 mM K-HEPES, 0.1% sodium deoxycholate, 1% Triton X-100, pH 7.5) with protease inhibitor cocktail (Sigma) at a concentration of 100 µl/100 ml starting culture. Next, sonication was performed to get sheared chromatin fragments of an average size of 300–700 bp by SONICS Vibra cell sonicator. The soluble fraction of sheared chromatin was obtained by centrifuging the sonicated solution at 13000 rpm for 15 min at 4°C.

#### Total DNA (T)

About 1/10^th^ of total soluble chromatin was processed separately as total DNA (whole cell lysate). Equal volume of elution buffer I (50 mM Tris·HCl, pH 8/10 mM EDTA/1% SDS) was added to the chromatin solution separated for Total DNA, and incubated at 65°C overnight to reverse crosslinking. TE (1×, 10 mM Tris·HCl, pH 8, 1 mM EDTA,) was added to the starting material to make final SDS concentration 0.5%. Sample was treated with RNase A (Sigma # R4875) and Proteinase K (NEB Cat # P8102). The DNA was extracted with an equal volume of phenol/chloroform/isoamyl alcohol (25∶24∶1) in the presence of 0.4 M LiCl and precipitated with ethanol for 15 min at room temperature. It was spun at 16,000× *g* for 20 min at room temperature. The DNA pellet was washed with 70% ethanol and resuspended in TE.

#### Immunoprecipitated material (IP)

Rest of the soluble chromatin solution was diluted 5.7-fold with IP dilution buffer (167 mM NaCl, 1.1 mM EDTA, 1.1% Triton X-100, 167 mM Tris–HCl, pH 8.0) and divided equally in two tubes. Rabbit anti-CaCse4p antibody was added to a final concentration of 4 µg/ml in one tube (+Ab) and no antibody (−Ab) was added to the other. The tubes were slowly rotated overnight at 4°C. A slurry of Protein A-Sepharose beads (50 µl per ml of 50% slurry in TE) (Sigma) was added and the tube was again rotated overnight at 4°C. Next beads were sequentially washed twice in 12 ml of extraction buffer, and once each in 12 ml of extraction buffer+500 mM NaCl, LiCl wash buffer (10 mM Tris-HCl, pH 8, 250 mM LiCl, 1 mM Igepal CA-630, 0.5% sodium deoxycholate/1 mM EDTA) and TE. Beads were subjected to elution of IP complexes in elution buffer I (1/10 volume of IP dilution buffer) at 65°C for 15 min, and then centrifuged at 4000 rpm for 5 min. Supernatant was collected and beads were washed a second time with elution buffer II (10 mM Tris-HCl, pH 8/1 mM EDTA/0.67% SDS) for 5 min at 65°C and the supernatant was collected as above. Pooled eluates were incubated overnight at 65°C to reverse crosslinking. To purify DNA, the SDS concentration was diluted to 0.5% with TE, and the reaction was treated with RNase A, followed by Proteinase K. The 4 M LiCl was added to a final concentration of 0.4 M and the DNA was extracted with an equal volume of phenol/chloroform/isoamyl alcohol (25∶24∶1). Aqueous layer was precipitated in 100% ethanol overnight at −20°C. DNA was recovered by centrifugation at 16,000× *g* for 45 min at 4°C, washed with 70% ethanol, spun for 15 min, and the DNA pellet was dried for 30 min. The recovered DNA was resuspended in TE.

ChIP assays with anti-Myc antibodies in J123 (MycMIF2) or anti-Prot-A antibodies were performed using the same protocol except for the following modification. J123 cells were crosslinked with 1% formaldehyde for 45 min. The protein enrichment on a specific DNA sequence was determined by specific PCR primers (Table S2).

### Subcellular immunolocalization

Indirect immunofluoroscence was performed using protocol described previously [49]. Asynchronous and exponentially growing culture was fixed using 1 ml 37% formaldehyde per 10 ml culture for 1 h. Fixed cells were washed and resuspended in 0.1 M Phosphate buffer (pH 6.4). Next, 70–80% spheroplasting was achieved using lyticase (Sigma) and β-mercaptoethanol. Spheroplasts were pelleted down gently at low speed and resuspended in PBS. Teflon coated slide was incubated with polylysine (1 mg/ml) for 5 min, washed with water and dried. Next 15 µl of fixed cells were placed onto each well and incubated for 5 min. Cells attached to the slides were fixed in ice cold methanol (−20°C) for 6 min and ice-cold acetone (−20°C) for 30 seconds. Blocking was performed with 2% skim milk in PBS for 30 min. Subsequently cells were incubated with primary antibodies for 1 h and washed with PBS four times. Subsequently, secondary antibodies were added onto each well and incubated 1 h in a dark humid chamber. Finally the slide was washed four times with phosphate buffered saline (PBS). DAPI solution (50 ng/ml in 70% glycerol) was added on to each well and coverslip and slide were sealed together. Co-immunolocalization experiments were performed using the same protocol.

### Image analysis

Images were captured using Carl Zeiss confocal laser scanning microscope (LSM 510 META) using LSM 5 Image Examiner. Three dimentional (3D) images were generated using LSM 3D rendering software (Figure 6 and Figure 12) (Carl Zeiss, Germany). Images were rotated in 3D and snapshots were taken from three different rotational angles (a-a″, b-b′, c-c″) in Figure 6. Images were susequently processed in Adobe Photoshop.

### Western blot analysis

Wild-type or conditional mutant strains were grown under inducing and repressed conditions for 8 h. Protein extracts were made by disrupting the cells in RIPA buffer (300 mM NaCl, 50 mM Tris-HCl pH 8.0, 5 mM EDTA pH 8.0, 0.5% Triton-X) using glass beads (Sigma cat # G8772). The lysate were subjected to electrophoresis using 12% SDS PAGE and transferred to nitrocellulose membrane for 1 h at 20 V by semi-dry method. Proper transfer was checked by Ponceau S staining. Membranes were blocked with 5% skim milk for 1 h followed by incubation with primary antibodies in 5% skim milk overnight at 4°C. Membranes were washed five times with PBS+0.05% Tween and incubated with secondary antibodies in 5% skim milk for 2 h. Membranes were washed five times with 1× PBS+0.05% Tween and developed by VersaDoc (Bio-Rad) or exposed to X-ray films. Quantification of the western blots was performed using the Quantity one software (Bio-Rad).

### Antibodies

Primary antibodies used for immunolocalization studies were as follows- affinity purified rabbit anti-Dad2 antibodies-1∶50 dilution, affinity purified rabbit anti-CENP-A antibodies - 1∶500 dilution [48], mouse anti-Myc -1∶50 dilution (Calbiochem, Cat # OP10L), rabbit anti-Prot A- 1∶1500 (Sigma Cat # P2921), rat anti-tubulin (Invitrogen, Cat # YOL1/34) - 1∶100 dilution. The fluorescent secondary antibodies for immunolocalization were obtained from Invitrogen and used at dilution 1∶500 for Alexa Fluor goat anti-rabbit IgG 568 (Cat #A11011) , 1∶100 for Alexa Fluor goat anti-rat IgG 488 (Cat # A11006) and 1∶500 for Alexa Fluor anti -mouse 488 (Cat # A11001).

Primary antibodies used for western blot analysis were rabbit anti-CENP-A (1∶500), mouse anti-PSTAIRE (1∶2000, Sigma Cat # P7962), rabbit anti-Prot A (1∶ 5000, Sigma Cat # P2921) and rabbit anti-Dad2 (1∶500, unpurified sera) antibodies. Secondary antibodies used were anti-rabbit HRP conjugated (1∶2000, Bangalore Genei Cat # 105499), anti-mouse HRP conjugated (1∶2000, Bangalore Genei, Cat # HP06).

## Supporting Information

Figure S1 Depletion of an outer kinetochore protein leads to reduced levels of CENP-A at the kinetochore in *C. albicans*. Wild-type, and conditional mutant J106 (*MET3*pr*SPC19/spc19*) or J108 (*PCK1*pr*DAD2/dad2*) were grown for 8 h under non-permissive conditions to deplete Spc19 or Dad2 respectively. These cells were fixed and stained with DAPI and anti-Cse4 antibodies. CENP-A/Cse4 signals, visible in wild-type cells, were undetected in Spc19 or Dad2 depleted cells. Bars, 5 µm.
(JPG)

Figure S2 CENP-A levels at kinetochores are independent of spindle integrity. Intensity of Cse4-GFP/KT was measured in untreated (DMSO) or nocodazole (NOC) treated 10118 (*CSE4:GFP:CSE4/cse4*) cells and plotted. No significant change in GFP intensity was observed between NOC treated cells and untreated cells.
(JPG)

Figure S3 The kinetochore cluster is disintegrated in absence of an essential kinetochore protein. (A) Dad2-depleted J108 (*PCK1*pr*DAD2/dad2*) cells grown for 5 h under non-permissive conditions were fixed and stained with DAPI and anti-Cse4 antibodies. These Dad2-depleted cells exhibited multiple weak Cse4 signals per nucleus suggesting that clustered KTs were in the process of disintegration. (B) Parent YJB10695 (*MTW1GFP/MTW1*) or *ask1* conditional mutant J120 (*MET3*pr*ASK1/ask1 MTW1GFP/MTW1*) cells grown under non-permissive conditions of the *MET3* promoter (+Cys +Met) for 5 h exhibited clustered or declustered Mtw1GFP signals in presence or absence of Ask1 respectively. Bars, 5 µm.
(JPG)

Figure S4 Kinetochore disintegration precedes kinetochore collapse. Levels of GFP-tagged Mtw1 (a middle KT protein) under gradual repression of CENP-A/Cse4 (an inner KT protein; upper panels) or Ask1 (an outer KT protein; lower panels) was monitored at indicated time after shift to non-permissive medium for CENP-A/Cse4 or Ask1 expression. In each case GFP (left) and GFP+DIC (right) images are shown. Bar, 5 µm.
(JPG)

Figure S5 CENP-A is unstable in absence of an essential kinetochore protein. (A) J102 (*MET3*pr*DAM1/dam1*) or J104 (*MET3*pr*ASK1/ask1*) expressing Dam1 or Ask1 from the *MET3* promoter and CAMB2 (*PCK1*pr*MIF2/mif2*) or J106 (*MET3*pr*SPC19/spc19*) expressing Mif2 or Spc19 from the *PCK1* promoter were grown overnight in inducing media and then transferred to repressing media for 8 h. Western blot analysis was performed using anti-Cse4 and anti-PSTAIRE antibodies with cell lysates prepared from 0 h and 8 h of growth in repressing media. CENP-A/Cse4 (right panels) protein levels showed a significant decrease when each of Dam1, Ask1, CENP-C/Mif2 or Spc19 was depleted.
(JPG)

Figure S6 Site-directed mutagenesis to create CENP-A/Cse4 mutant Cse4^7R^ in *C. albicans*. All seven lysine residues were changed to arginine residues in *CSE4* ORF using the site-directed mutagenesis kit (Stratagene). Incorporation of all the changes was confirmed by sequencing of relevant regions from wild-type J128 (*CSE4-TAP(URA3)/cse4::hisG*, top panel) or J129 (*CSE4^7R^-TAP(URA3)/ cse4::hisG*, bottom panel) strain. Changes K189-R189 and K196- R196 were confirmed by the reverse primer.
(JPG)

Table S1 *C. albicans* strains used in this study.
(DOC)

Table S2 Primers used in this study.
(DOC)
