# Supplementary figures and images for: A Coordinated Interdependent Protein Circuitry Stabilizes the Kinetochore Ensemble to Protect CENP-A in the Human Pathogenic Yeast Candida albicans
Source: PLoS Genet. 2012 Apr 19;8(4):e1002661. doi: 10.1371/journal.pgen.1002661 (PMC3334883; doi:10.1371/journal.pgen.1002661)

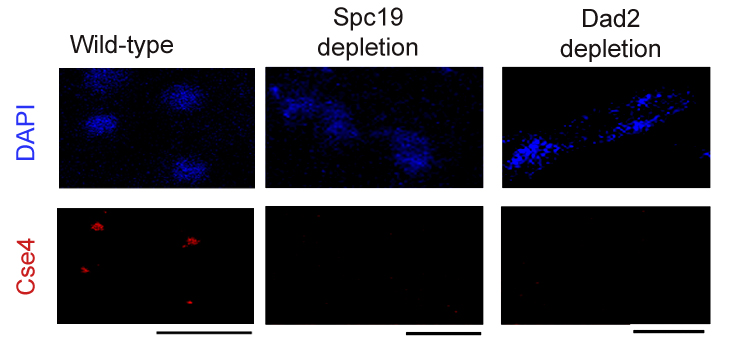

Supplement: Figure S1 — Depletion of an outer kinetochore protein leads to reduced levels of CENP-A at the kinetochore in C. albicans. Wild-type, and conditional mutant J106 (MET3prSPC19/spc19) or J108 (PCK1prDAD2/dad2) were grown for 8 h under non-permissive conditions to deplete Spc19 or Dad2 respectively. These cells were fixed and stained with DAPI and anti-Cse4 antibodies. CENP-A/Cse4 signals, visible in wild-type cells, were undetected in Spc19 or Dad2 depleted cells. Bars, 5 µm. (JPG) [file pgen.1002661.s001.jpg]

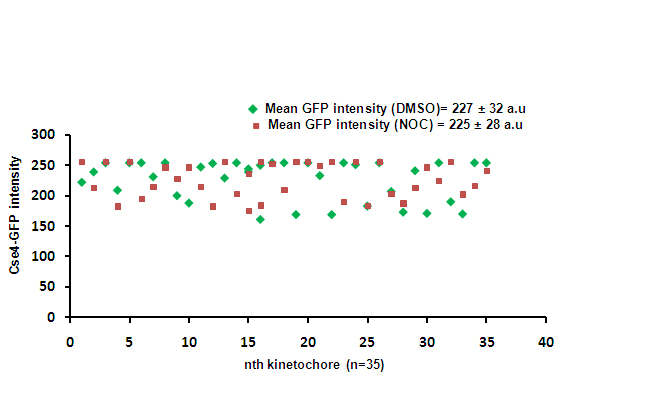

Supplement: Figure S2 — CENP-A levels at kinetochores are independent of spindle integrity. Intensity of Cse4-GFP/KT was measured in untreated (DMSO) or nocodazole (NOC) treated 10118 (CSE4:GFP:CSE4/cse4) cells and plotted. No significant change in GFP intensity was observed between NOC treated cells and untreated cells. (JPG) [file pgen.1002661.s002.jpg]

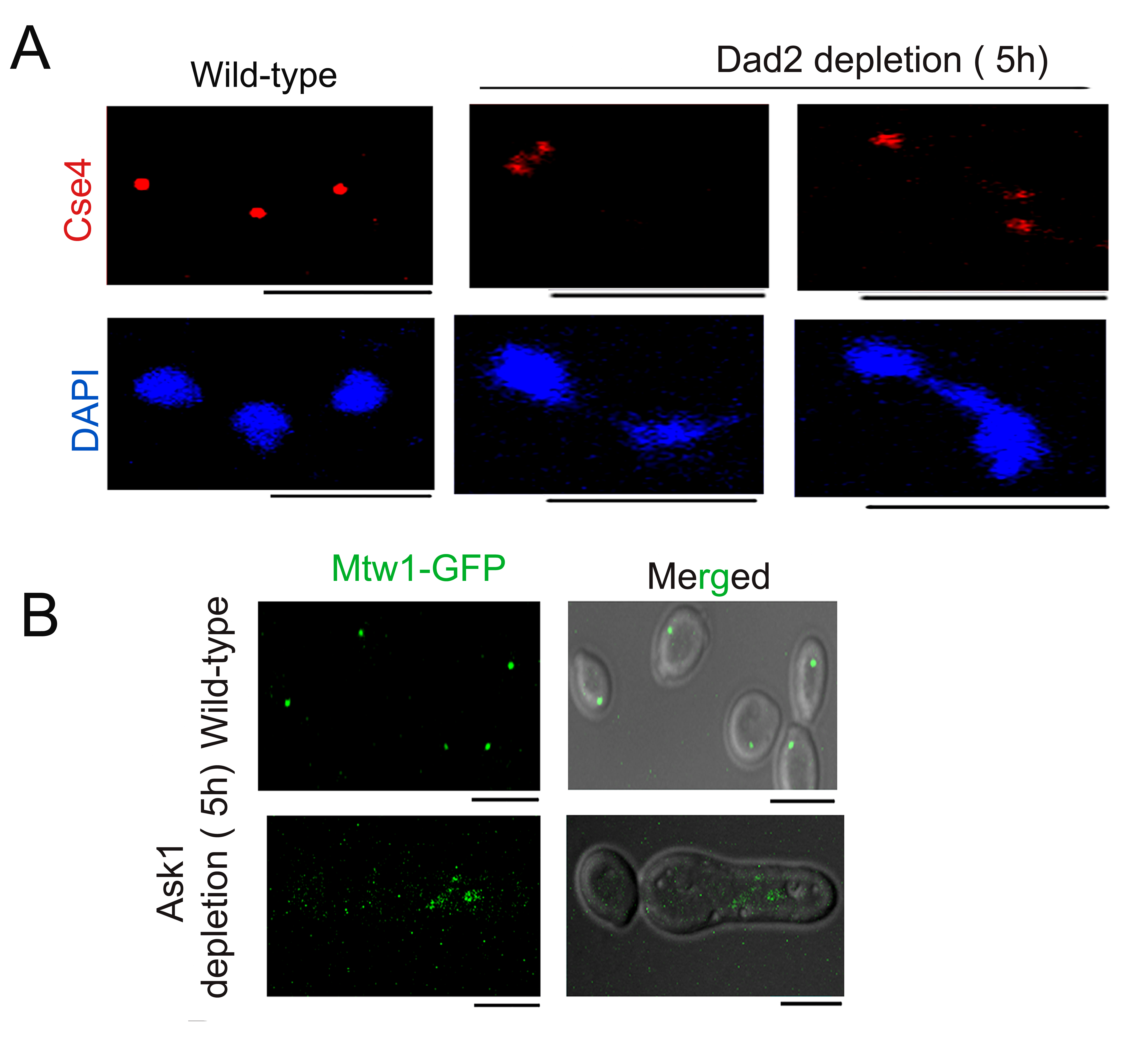

Supplement: Figure S3 — The kinetochore cluster is disintegrated in absence of an essential kinetochore protein. (A) Dad2-depleted J108 (PCK1prDAD2/dad2) cells grown for 5 h under non-permissive conditions were fixed and stained with DAPI and anti-Cse4 antibodies. These Dad2-depleted cells exhibited multiple weak Cse4 signals per nucleus suggesting that clustered KTs were in the process of disintegration. (B) Parent YJB10695 (MTW1GFP/MTW1) or ask1 conditional mutant J120 (MET3prASK1/ask1 MTW1GFP/MTW1) cells grown under non-permissive conditions of the MET3 promoter (+Cys +Met) for 5 h exhibited clustered or declustered Mtw1GFP signals in presence or absence of Ask1 respectively. Bars, 5 µm. (JPG) [file pgen.1002661.s003.jpg]

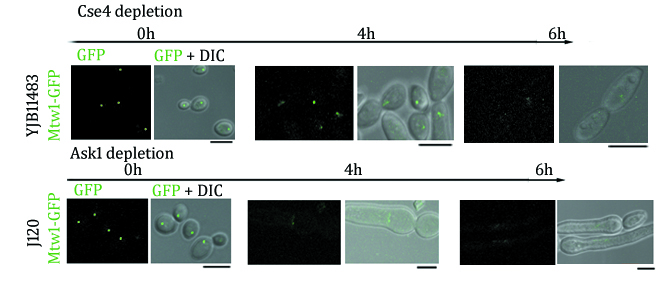

Supplement: Figure S4 — Kinetochore disintegration precedes kinetochore collapse. Levels of GFP-tagged Mtw1 (a middle KT protein) under gradual repression of CENP-A/Cse4 (an inner KT protein; upper panels) or Ask1 (an outer KT protein; lower panels) was monitored at indicated time after shift to non-permissive medium for CENP-A/Cse4 or Ask1 expression. In each case GFP (left) and GFP+DIC (right) images are shown. Bar, 5 µm. (JPG) [file pgen.1002661.s004.jpg]

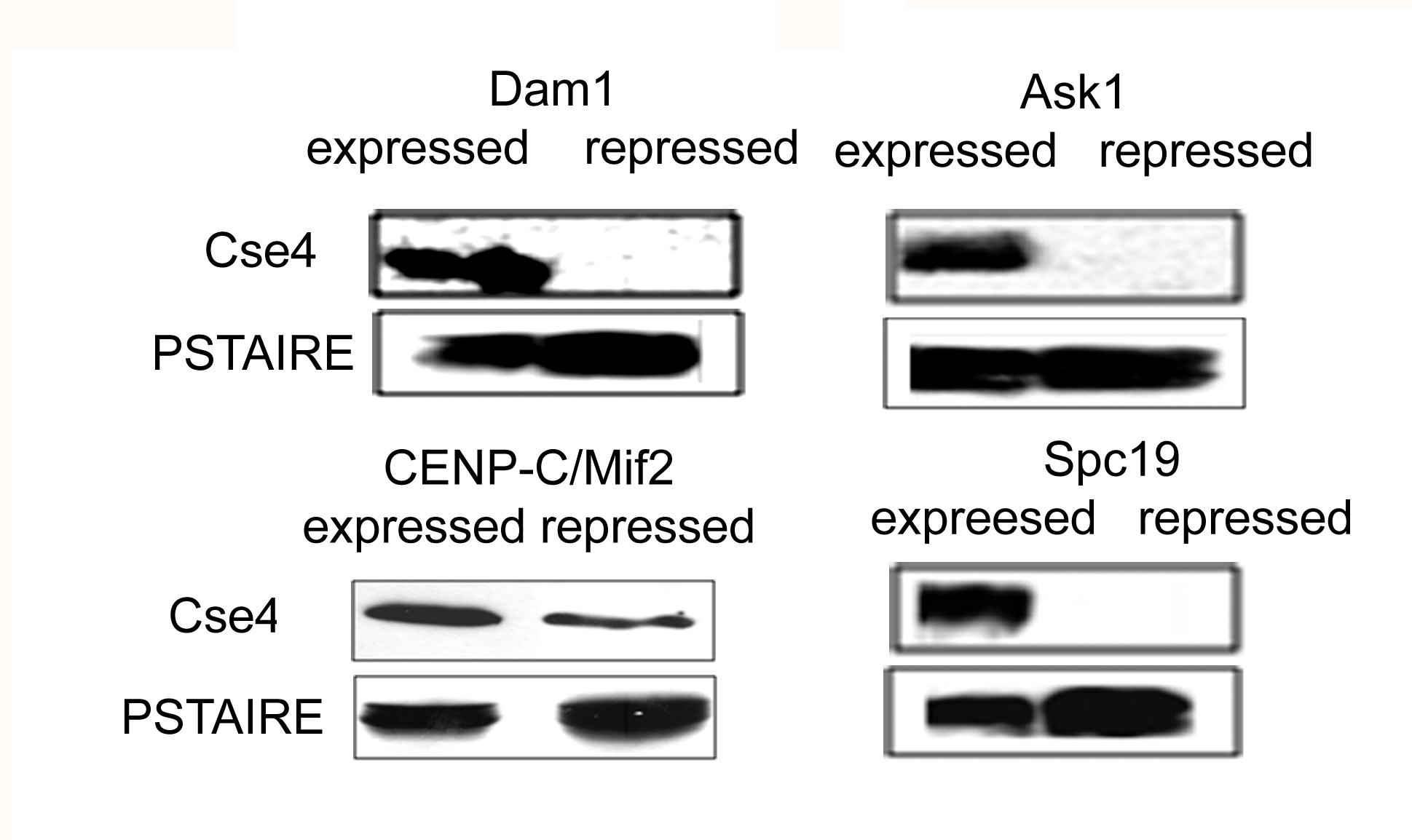

Supplement: Figure S5 — CENP-A is unstable in absence of an essential kinetochore protein. (A) J102 (MET3prDAM1/dam1) or J104 (MET3prASK1/ask1) expressing Dam1 or Ask1 from the MET3 promoter and CAMB2 (PCK1prMIF2/mif2) or J106 (MET3prSPC19/spc19) expressing Mif2 or Spc19 from the PCK1 promoter were grown overnight in inducing media and then transferred to repressing media for 8 h. Western blot analysis was performed using anti-Cse4 and anti-PSTAIRE antibodies with cell lysates prepared from 0 h and 8 h of growth in repressing media. CENP-A/Cse4 (right panels) protein levels showed a significant decrease when each of Dam1, Ask1, CENP-C/Mif2 or Spc19 was depleted. (JPG) [file pgen.1002661.s005.jpg]

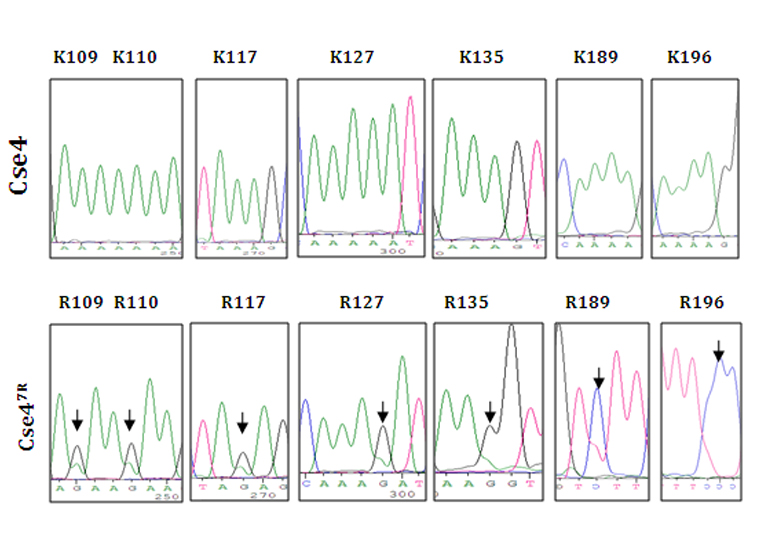

Supplement: Figure S6 — Site-directed mutagenesis to create CENP-A/Cse4 mutant Cse47R in C. albicans. All seven lysine residues were changed to arginine residues in CSE4 ORF using the site-directed mutagenesis kit (Stratagene). Incorporation of all the changes was confirmed by sequencing of relevant regions from wild-type J128 (CSE4-TAP(URA3)/cse4::hisG, top panel) or J129 (CSE47R-TAP(URA3)/ cse4::hisG, bottom panel) strain. Changes K189-R189 and K196- R196 were confirmed by the reverse primer. (JPG) [file pgen.1002661.s006.jpg]
