# Supplementary material for: A Coordinated Interdependent Protein Circuitry Stabilizes the Kinetochore Ensemble to Protect CENP-A in the Human Pathogenic Yeast Candida albicans
Source: PLoS Genet. 2012 Apr 19;8(4):e1002661. doi: 10.1371/journal.pgen.1002661 (PMC3334883; doi:10.1371/journal.pgen.1002661)
Supplement: Table S1 — C. albicans strains used in this study. (DOC) [file pgen.1002661.s007.doc]

**Table S1.** ***C. albicans* strains used in this study.**

| **Strain name** | ***Genotype*** | **Reference** |
| --- | --- | --- |
| **BWP17** | *Δura3::imm434/ Δura3::imm434 Δhis1::hisG/ Δhis1::hisG Δarg4::hisG/ Δarg4::hisG* | [1] |
| **SN148** | *ura3Δ-iro1Δ::imm434/ ura3Δ-iro1Δ::imm434, his1Δ/his1Δ, arg4Δ/arg4Δ., leu2Δ/leu2Δ* | [2] |
| **CAMB1** | *Δura3::imm434/ Δura3::imm434 Δhis1::hisG/ Δhis1::hisG Δarg4::hisG/ Δarg4::hisG* ***MIF2/ mif2::PCK1*pr*12XMycMIF2 (URA3)*** | [3] |
| **CAMB2** | *Δura3::imm434/ Δura3::imm434 Δhis1::hisG/ Δhis1::hisG Δarg4::hisG/ Δarg4::hisG* ***mif2::PCK1*pr*12XMycMIF2 (URA3)/ mif2::HIS1*** | [3] |
| **CAKS2b** | *Δura3::imm434/ Δura3::imm434 Δhis1::hisG/ Δhis1::hisG Δarg4::hisG/ Δarg4::hisG* ***CSE4/******cse4::hisG*** | [4] |
| **CAKS3b** | *Δura3::imm434/ Δura3::imm434 Δhis1::hisG/ Δhis1::hisG Δarg4::hisG/ Δarg4::hisG* ***PCK1*pr*CSE4(URA3)/******cse4::hisG*** | [4] |
| **10118** | *ura3Δ-iro1Δ::imm434/ ura3Δ-iro1Δ::imm434, his1Δ/his1Δ, arg4Δ/arg4Δ.,* ***CSE4:GFP:CSE4/ cse4::dpl200-URA3*** | [5] |
| **YJB10695** | *Δura3*::*imm434/Δura3*::*imm434 Δhis1*::*hisG/Δhis1*::*hisG Δarg4*::*hisG/Δarg4*::*hisG,* ***MTW1-*GFP (*NAT*)/*****MTW1*** | [6] |
| **YJB11990** | *Δura3*::*imm434/Δura3*::*imm434 Δhis1*::*hisG/Δhis1*::*hisG Δarg4*::*hisG/Δarg4*::*hisG* ***PCK1*pr*CSE4 (URA)3/CSE4 NAT1-MET3*pr*DAM1/dam1::HIS1*** | [7] |
| **YJB11483** | *Δura3*::*imm434/Δura3*::*imm434 Δhis1*::*hisG/Δhis1*::*hisG Δarg4*::*hisG/Δarg4*::*hisG,* ***PCK1*pr*CSE4(URA3)/cse4*::*hisG, MTW1/MTW1-*GFP*(NAT)*** | [6] |
| **RM1000AH** | Δ*ura3::*imm434/ Δ*ura3::*imm434 Δ*his1::*hisG/ Δ*his1::*hisG arg4::***HIS1*/*ARG4*** | [3] |
| **CAKS12** | *Δura3::imm434/Δura3::imm434 Δhis1::hisG/Δhis1::hisG Δarg4::hisG/Δarg4::hisG,****PCK1*pr*MTW1(URA3)/mtw1::HIS1*** | [6] |
| **YJB12326** | *Δura3*::*imm434/Δura3*::*imm434 Δhis1*::*hisG/Δhis1*::*hisG Δarg4*::*hisG/Δarg4*::*hisG,* ***PCK1*pr*CSE4(URA3)/CSE4 NAT1-MET3*pr*NUF2/nuf2::HIS1*** | [7] |
| **YJB12289** | *Δura3*::*imm434/Δura3*::*imm434 Δhis1*::*hisG/Δhis1*::*hisG Δarg4*::*hisG/Δarg4*::*hisG* ***PCK1*pr*CSE4(URA3)/CSE4 NAT1-MET3*pr*DAM1/dam1::HIS1 NUF2-GFP-ARG4/NUF2*** | [7] |
| **J102** | *ura3Δ-iro1Δ::imm434/ ura3Δ-iro1Δ::imm434, his1Δ/his1Δ, arg4Δ/arg4Δ., leu2Δ/leu2Δ* ***MET3*pr*DAM1(URA3)/dam1::HIS1*** | [8] |
| **J104** | *ura3Δ-iro1Δ::imm434/ ura3Δ-iro1Δ::imm434, his1Δ/his1Δ, arg4Δ/arg4Δ., leu2Δ/leu2Δ* ***MET3*pr*ASK1(URA3)/ask1::HIS1*** | [8] |
| **J106** | *ura3Δ-iro1Δ::imm434/ ura3Δ-iro1Δ::imm434, his1Δ/his1Δ, arg4Δ/arg4Δ., leu2Δ/leu2Δ* ***MET3*pr*SPC19(URA3)/spc19::HIS1*** | [8] |
| **J108** | *Δura3::imm434/ Δura3::imm434 Δhis1::hisG/ Δhis1::hisG Δarg4::hisG/ Δarg4::hisG* ***PCK1*pr*DAD2(URA3)/ dad2::HIS1*** | [8] |
| **J119** | *ura3Δ-iro1Δ::imm434/ ura3Δ-iro1Δ::imm434, his1Δ/his1Δ, arg4Δ/arg4Δ., leu2Δ/leu2Δ* ***ASK1/ask1::HIS1 MTW1/MTW1GFP*** | This study |
| **J120** | *ura3Δ-iro1Δ::imm434/ ura3Δ-iro1Δ::imm434, his1Δ/his1Δ, arg4Δ/arg4Δ., leu2Δ/leu2Δ* ***MET3*pr*ASK1(CdARG4)/ask1::HIS1 MTW1/MTW1GFP*** | This study |
| **J121** | *ura3Δ-iro1Δ::imm434/ ura3Δ-iro1Δ::imm434, his1Δ/his1Δ, arg4Δ/arg4Δ., leu2Δ/leu2Δ* ***MET3*pr*DAM1(CdARG4)/dam1::HIS1 MTW1GFP/ MTW1*** | This study |
| **J122** | *ura3Δ-iro1Δ::imm434/ ura3Δ-iro1Δ::imm434, his1Δ/his1Δ, arg4Δ/arg4Δ., leu2Δ/leu2Δ* ***/MET3*pr*DAM1(CdARG4)/dam1::HIS1 MTW1GFP/ MTW1*** | This study |
| **J123** | *ura3Δ-iro1Δ::imm434/ ura3Δ-iro1Δ::imm434, his1Δ/his1Δ, arg4Δ/arg4Δ., leu2Δ/leu2Δ* ***MET3*pr*DAM1(URA3)/dam1::HIS1 MIF2/PCK1*pr*12XMYCMIF2(NAT)*** | This study |
| **J124** | *ura3Δ-iro1Δ::imm434/ ura3Δ-iro1Δ::imm434, his1Δ/his1Δ, arg4Δ/arg4Δ., leu2Δ/leu2Δ* ***/MET3*pr*ASK1(URA3)/ask1::HIS1 MIF2/2PCK1*pr*12XMYCMIF2(NAT)*** | This study |
| **J125** | *Δura3::imm434/ Δura3::imm434 Δhis1::hisG/ Δhis1::hisG arg4::****HIS1*/*ARG4 MIF2*/ *mif2*::*PCK1_pr_12XMycMIF2* (*NAT1*)** | This study |
| **J126** | *ura3Δ-iro1Δ::imm434/ ura3Δ-iro1Δ::imm434, his1Δ/his1Δ, arg4Δ/arg4Δ.,* ***DAM1/dam1::HIS1 cse4::dpl200-URA3/CSE4:GFP:CSE4*** | This study |
| **J127** | *ura3Δ-iro1Δ::imm434/ ura3Δ-iro1Δ::imm434, his1Δ/his1Δ, arg4Δ/arg4Δ.,* ***MET3*pr*DAM1(CdARG4)/dam1::HIS1 cse4::dpl200-URA3/CSE4:GFP:CSE4*** | This study |
| **J128** | *Δura3::imm434/ Δura3::imm434 Δhis1::hisG/ Δhis1::hisG Δarg4::hisG/ Δarg4::hisG* ***CSE4-TAP(URA3)/******cse4::hisG*** | This study |
| **J129** | *Δura3::imm434/ Δura3::imm434 Δhis1::hisG/ Δhis1::hisG Δarg4::hisG/ Δarg4::hisG* ***CSE4^7R^-TAP(URA3)/******cse4::hisG*** | This study |
| **J130** | *ura3Δ-iro1Δ::imm434/ ura3Δ-iro1Δ::imm434, his1Δ/his1Δ, arg4Δ/arg4Δ.,* ***MET3*pr*DAM1(URA3)/dam1::HIS1 CSE4 / CSE4-TAP*** | This study |
| **J131** | *ura3Δ-iro1Δ::imm434/ ura3Δ-iro1Δ::imm434, his1Δ/his1Δ, arg4Δ/arg4Δ.,* ***MET3*pr*DAM1(URA3)/dam1::HIS1 CSE4 / CSE4^7R^-TAP*** | This study |

**References**

1. Wilson RB, Davis D, Mitchell AP (1999) Rapid hypothesis testing with Candida albicans through gene disruption with short homology regions. J Bacteriol 181: 1868-1874.

2. Noble SM, Johnson AD (2005) Strains and strategies for large-scale gene deletion studies of the diploid human fungal pathogen Candida albicans. Eukaryotic Cell 4: 298-309.

3. Sanyal K, Baum M, Carbon J (2004) Centromeric DNA sequences in the pathogenic yeast Candida albicans are all different and unique. Proc Natl Acad Sci U S A 101: 11374-11379.

4. Padmanabhan S, Thakur J, Siddharthan R, Sanyal K (2008) Rapid evolution of Cse4p-rich centromeric DNA sequences in closely related pathogenic yeasts, Candida albicans and Candida dubliniensis. Proc Natl Acad Sci U S A 105: 19797-19802.

5. Joglekar AP, Bouck D, Finley K, Liu X, Wan Y, et al. (2008) Molecular architecture of the kinetochore-microtubule attachment site is conserved between point and regional centromeres. J Cell Biol 181: 587-594.

6. Roy B, Burrack LS, Lone MA, Berman J, Sanyal K (2011) CaMtw1, a member of the evolutionarily conserved Mis12 kinetochore protein family, is required for efficient inner kinetochore assembly in the pathogenic yeast Candida albicans. Mol Microbiol.

7. Burrack LS, Applen SE, Berman J (2011) The requirement for the Dam1 complex is dependent upon the number of kinetochore proteins and microtubules. Curr Biol 21: 889-896.

8. Thakur J, Sanyal K (2011) The essentiality of the fungus-specific Dam1 complex is correlated with a one-kinetochore-one-microtubule interaction present throughout the cell cycle, independent of the nature of a centromere. Eukaryot Cell 10: 1295-1305.
