# Supplementary material for: A Coordinated Interdependent Protein Circuitry Stabilizes the Kinetochore Ensemble to Protect CENP-A in the Human Pathogenic Yeast Candida albicans
Source: PLoS Genet. 2012 Apr 19;8(4):e1002661. doi: 10.1371/journal.pgen.1002661 (PMC3334883; doi:10.1371/journal.pgen.1002661)
Supplement: Table S2 — Primers used in this study. (DOC) [file pgen.1002661.s008.doc]

**Table S2. Primers used in this study.**

| **Primer name** | **Sequence** | **Description** |
| --- | --- | --- |
| For generation of conditional mutants in the background of strains expressing various epitope tagged KT proteins | | |
| DamdelHis1 | GAG ATC TCT TAT TAT TTG ATT TCT TTT TCT GCA AAT TAA CAA CCT TTC AGG AGA ATG TCC TCA TCT AAA CCA GTT ACC CCT AGG AAT TCC GGG GAT CCT GGA GGA TGA GGA G | Forward long primer for the replacement of first allele of *DAM1* with *HIS1* |
| DamdelHis2 | CAA AAC AAG TCT GTT GAA CAA GTG AAA TAC GAA TTA AAA CTA AAA CTC ATA TAC GGG GGA TAA GTA TAT ATA TAT ATA TAT AAC GAA TTC CGG AAT ATT TAT GAG AAA CTA TCA C | Reverse long primer for the replacement of first allele of *DAM1*with *HIS1* |
| DamdelHis3 | CAA ACA AAA TAG TAA AAA TTA TTG AGA TCT CTT ATT ATT TG | Forward short primer to amplify cassette cloned in pBluescript using DamdelHis1 and DamdelHis2 for the replacement of first allele of *DAM1* with *HIS1* |
| DamdelHis4 | CTT TTA ATG ATT GCC TCC CAA CAA AAC AAG TCT GTT GAA CAA G | Reverse short primer to amplify cassette cloned in pBluescript using DamdelHis1 and DamdelHis2 for the replacement of first allele of *DAM1* with *HIS1* |
| Dam1probe5' | CAA AGA CCT GTT GAT AGA TTG TTG ACT AC | Forward primer for the confirmation of the replacement of deletion of *DAM1* with *HIS1* |
| Ask1His1 | CAC GCA AAA AAG AAA AAC CAC AAC AAA TCA GAA AAC AAC AAC AAC ATC ATC ATA TCA ACA TTA ACC AAC TTT CTG CTG CAT CAA ATG AAT TCC GGG GAT CCT GGA GGA TGA GGA G | Forward long primer for the replacement of first allele of *ASK1*with *HIS1* |
| Ask1His2 | CTA CGA TGA TTT CAT TAC TAA TAT TAT ATT TTT ACA TTA CTA CCA ACT GTT GAT GTC TAT TGA CTA CGT AGT TAT GTT TTC CTC CGA ATT CCG GAA TAT TTA TGA GAA ACT ATC AC | Reverse long primer for the replacement of first allele of *ASK1*with *HIS1* |
| Ask1His3 | GCA CTA GTC GCA CAC ACA CCT ACA CGC AAA AAA GAA AAA C | Forward short primer to amplify cassette cloned in pBluescript using Ask1His1and Ask1His2 for the replacement of first allele of *ASK1*with *HIS1* |
| Ask1His4 | GGG AAG CTT CTT ACT TGA AAA ACT ACG ATG | Reverse short primer to amplify cassette cloned in pBluescript using Ask1His1and Ask1His2 for the replacement of first allele of *ASK1*with *HIS1* |
| Ask1HisProbe | CTC TGT GTA CCA TTT ATT AAA ACA ATC ATC | Forward primer for the confirmation of the replacement of deletion of *ASK1* with *HIS1* |
| Damet1 | CGG GAT CCG ATG TCC TCA TCT AAA CCA GTT AC | Forward primer with *BamH*I site at 5´ for placing *DAM1* under control of the *MET3* promoter |
| Damet2 | CGG GAT CCG ATC GTG GCT GTT GTT TAA TCT G | Reverse primer with *BamH*I site at 5´ for placing *DAM1* under control of the *MET3* promoter |
| AskMet1 | CGG GAT CCG ATG AAG AGA TAT TCA ATT GC | Forward primer with *BamH*I site at 5´ for placing *ASK1* under control of the *MET3* promoter |
| AskMet2 | CGGGATCCCTAGATTGTAGTCGTTGATGTTG | Reverse primer with *BamH*I site at 5´ for placing *ASK1* under control of the *MET3* promoter |
| URA3-1 | G GTC TTA GTG TTG ACT GTC | Reverse primer for the confirmation of integration of *MET3* containing Cassettes at *DAM1,ASKI* and *SPC19* loci |
| Mif2pckMycPst1F | AGA ACT GAC ACT GCA GGG ATC CAC TGT ATT CCA ATT TAA CAG AG | MycMif2 integration |
| Mif2 pck1SacIIR | GAG CCG CGG CTG TAA CTG GTG GTT TAA ATC CAT CC | MycMif2 integration |
| For site-directed mutagenesis | | |
| K109-110R | cgaacagtacctcgagtgagaagaagatatcgtccaggtact | Site directed mutagenesis of CSE4 to CSE4^7R^ |
| K109-110R_antisense | agtacctggacgatatcttcttctcactcgaggtactgttcg | Site directed mutagenesis of CSE4 to CSE4^7R^ |
| K117R | aaaaaagatatcgtccaggtactagagctttacgagagatcag | Site directed mutagenesis of CSE4 to CSE4^7R^ |
| K117R_antisense | ctgatctctcgtaaagctctagtacctggacgatatctttttt | Site directed mutagenesis of CSE4 to CSE4^7R^ |
| K127R | ttacgagagatcagacagtatcaaagatccactgatttattgattag | Site directed mutagenesis of CSE4 to CSE4^7R^ |
| K127R_antisense | ctaatcaataaatcagtggatctttgatactgtctgatctctcgtaa | Site directed mutagenesis of CSE4 to CSE4^7R^ |
| K135R | ccactgatttattgattagaaggttgccctttgcccg | Site directed mutagenesis of CSE4 to CSE4^7R^ |
| K135R_antisense | cgggcaaagggcaaccttctaatcaataaatcagtgg | Site directed mutagenesis of CSE4 to CSE4^7R^ |
| K189R | atgtgcgattcacgccagaagagtgactatcatgc | Site directed mutagenesis of CSE4 to CSE4^7R^ |
| K189R_antisense | gcatgatagtcactcttctggcgtgaatcgcacat | Site directed mutagenesis of CSE4 to CSE4^7R^ |
| K196R | GCC AGA AGA GTG ACT ATC ATG CAA AGG GAT ATA CAA TTA GCA AGA | Site directed mutagenesis of CSE4 to CSE4^7R^ |
| K196R_antisense | TCT TGC TAA TTG TAT ATC CCT TTG CAT GAT AGT CAC TCT TCT GGC | Site directed mutagenesis of CSE4 to CSE4^7R^ |
| For construction and integration of pCSE4 TAP and pCSE4^7R^ TAP | | |
| Cse4SacII-F | ATA ACC GCG GAT GGC AAG ACT TTC AGG AC | Construction of pBSCSE4 |
| Cse4SpeI-R | TGA TAC TAG TCG TGT TAC AAT ATC CAA GAC TGT CC | Construction of pBSCSE4 |
| Cse4DS-SpeI-F | TGA TAC TAG TCA CGA TTT GGA ATA GA | Construction of pCSE4 TAP and pCSE4^7R^ TAP |
| Cse4DS-SmaI-F | ATA TCC CGG GGC TTG TGG TTA CTT TTC G | Construction of pCSE4 TAP and pCSE4^7R^ TAP |
| Cse4 probe 5’ | GTA ATA TCA CGC GTA CTG C | Confirmatory PCR for confirmation of integration of pCSE4 TAP and pCSE4^7R^ TAP |
| For ChIP analysis | | |
| p20057-3 | GTCTTCGATTGCAGTGGCTCCTCAAT | Co-ordinates- Assembly 21 CaChrR-1745148-1745172 |
| p20057-4 | GCAACCACACAAACATGTGTCTCG | Co-ordinates- Assembly 21 CaChrR-1745145-1745168 |
| p2488-1 | CACTCTGACCAAATTCTCGTTTCC | Co-ordinates- Assembly 21 CaChr1-1567094 - 1567117 |
| p2488-2 | GCAACATCCGAGTAAGGTTTGTGG | Co-ordinates- Assembly 21 CaChr1-1566850 - 1566873 |
| CACH2F | ACTGCTGGGCTTGTGAAGTT | Co-ordinates- Assembly 21 CaChr2-1929703 - 1929723 |
| CACH2R | GAGTCACAGCCAAACACGAA | Co-ordinates- Assembly 21 CaChr2-1929971 - 1929951 |
| CACH3F | AGATATGACGGCGCTGTTG | Co-ordinates- Assembly 21 CaChr3-823849 - 823861 |
| CACH3R | CAAACATCAACCTCCCCAAT | Co-ordinates- Assembly 21 CaChr3-824148 - 814128 |
| CACH4R | CTCTAATAACGCTAATCAAGAGA | Co-ordinates- Assembly 21 CaChr4-1004002 - 1003979 |
| CACH4F | GCTCTTACAAAAGCCAATCCA | Co-ordinates- Assembly 21 CaChr4-1003913 - 1003934 |
| CEN5-168F | TAATACCTAATGCTCATTCTTCCG | Co-ordinates- Assembly 21 CaChr5-470830-470853 |
| CEN5-168R | ATTTTCATGGAAGAGGGGTTTCAT | Co-ordinates- Assembly 21 CaChr5-470686-470709 |
| p1873-1 | GTAGGTGAGCGTTCAGAAGTCTGC | Co-ordinates- Assembly 21 CaChr6-979483 - 979506 |
| p1873-2 | CCGGAAAACAACATGGGTCTTGAC | Co-ordinates- Assembly 21 CaChr6-979722 - 979745 |
| 2498-21 | CTG GTG CAA GAC CCT CAT AGA AGC | Co-ordinates- Assembly 21 CaChr7 - 427537 - 427560 |
| 2498-22 | CCT GAC ACT GTC GTT TCC CAT AGC | Co-ordinates -Assembly 21 CaChr7 - 427369 - 427392 |
| 2498-11 | CGT ACA GTT GCG CAC TCA TTC AGA TG | Co-ordinates- Assembly 21 CaChr7 - 444850 - 444875 |
| 2498-12 | CAG TCT GCC AAG TGT AAA ACT GAG G | Co-ordinates -Assembly 21 CaChr7 - 444584 - 444608 |
| CaLeu2-1 | GTGACCATGTCGGTACCGAAATTGTC | Co-ordinates- Assembly 21 CaChr7 -64195 - 64220 |
| CaLeu2-2 | CTTGTTCAGGACGAACAGTGCCAGTA | Co-ordinates- Assembly 21 CaChr7 -64415 - 64440 |
